# The ZnO‐SiO_2_ Composite Phase with Dual Regulation Function Enables Uniform Zn^2+^ Flux and Fast Zinc Deposition Kinetics Toward Zinc Metal Batteries

**DOI:** 10.1002/advs.202411995

**Published:** 2024-12-04

**Authors:** Dongfang Guo, Fengyu Li, Bin Zhang

**Affiliations:** ^1^ School of Physics and Microelectronics Zhengzhou University Zhengzhou 450001 China; ^2^ School of Physics and Laboratory of Zhongyuan Light Zhengzhou University Zhengzhou 450001 China

**Keywords:** anode, aqueous batteries, dendritic‐free, zinc ion

## Abstract

As an important candidate for rechargeable energy storage devices, the large‐scale development of aqueous zinc ion batteries has been hindered by hydrogen evolution and uncontrollable dendrites of metal anodes. A novel ZnO‐SiO_2_ composite interface phase (Zn@ZSCP) with a double protective effect based on in situ synthesis by hydrothermal method is used to improve these difficulties. The hydrophilic SiO_2_ layer is beneficial to the dissolution of hydrated zinc ions and reduces the nucleation barrier during zinc deposition, while the stable ZnO layer helps to adjust the electric field distribution on the surface of the metal anode to further induce uniform zinc nucleation. The cycle life of the Zn@ZSCP||Zn@ZSCP symmetric battery based on this innovative interface phase modification is up to 2500 h. Even at a high current density of 8 mA cm^−2^, the symmetric battery still has a stable cycle life of more than 2000 h. The zinc‐iodine full battery based on Zn@ZSCP anode and low‐cost biomass‐derived porous carbon exhibits an excellent specific capacity and outstanding cycle stability. This simple and reasonable battery structure design not only improves the practicability of aqueous zinc ion batteries to a certain extent but also helps to develop more efficient and environmentally friendly zinc metal batteries.

## Graphical abstract

A novel ZnO‐SiO2 composite interface phase (ZSCP) is used to suppress the side reactions and dendrites of metal anodes in aqueous zinc ion batteries. The stable SiO_2_ layer and ZnO layer not only effectively adjust the electric field distribution on the anode surface to induce uniform zinc nucleation behavior, but also accelerate the desolvation process of zinc ions.

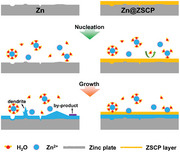

## 1 Introduction

The rapid development of industry and the continuous rise in energy demand have led to high expectations for advanced energy storage technologies.^[^ ^1^ ^]^ Lithium‐ion batteries occupy a dominant position in the consumer electronics market due to their excellent performance.^[^ ^2^ ^]^ However, rechargeable lithium‐ion batteries are demanding in terms of maintenance costs, assembly processes, and electrolyte selection, which has prompted the discovery of alternative energy storage technologies.^[^ ^3^ ^]^ The mild aqueous zinc‐ion battery has attracted much attention as a candidate for large‐scale energy storage due to its simple manufacturing process, low cost, high safety, and impressive theoretical volume energy density of 5855 mAh cm^−3^.^[^ ^4^ ^]^ However, the long‐term stability of aqueous zinc ion batteries is usually limited by zinc metal anodes. Zinc metal anodes will face a series of challenges such as corrosion, hydrogen evolution, and uncontrollable growth of zinc dendrites during cycling. In particular, irregular zinc dendrite growth caused by uneven zinc stripping/deposition behavior will not only reduce the overall energy storage efficiency of the battery and damage the battery life but also may puncture the separator and lead to a short circuit of the battery.^[^ ^5^ ^]^ To improve these defects, various strategies such as diaphragm modification,^[^ ^6^ ^]^ electrolyte optimization,^[^ ^7^ ^]^ interface modification engineering,^[^ ^8^ ^]^ and zinc alloy anode^[^ ^9^ ^]^ have been tried.

Among these strategies, interface modification engineering based on an artificial protective layer is valued due to its clear mechanism and simple operation.^[^ ^10^ ^]^ The optimization mechanism of the artificial modification layer mainly involves the following aspects: a) Promote the orderly flow of zinc ions; b) inhibiting the corrosion reaction between water molecules and metal anodes; c) Ensure favorable electric field distribution; d) Provide uniform nucleation sites. In summary, the fundamental purpose of the interface modification project is to significantly improve the stability and service life of aqueous zinc ion batteries to promote the sustainable and reliable development of the final energy storage system. Both conductive and non‐conductive modification layers can be used as artificial protective layers at the anode interface of zinc ion batteries.^[^ ^11^ ^]^ The conductive modified layer can be used as a new electrochemical reaction interface in the zinc deposition process, and the expansion of the effective active region can effectively limit the proliferation of zinc dendrites. At the same time, some modification layers can inhibit the overpotential by increasing the hydrogen evolution reaction (HER) to inhibit the anode side reaction.^[^ ^12^ ^]^ On the other hand, the non‐conductive modified layer does not change the reaction interface of the zinc anode but can be used to uniformly regulate the electric field strength of the anode surface, which helps to sort out the orderly diffusion of zinc ions at the interface to minimize the “tip effect” of the anode.^[^ ^13^ ^]^ In addition, the introduction of the modified layer can isolate the direct contact between the zinc metal surface and water molecules, thereby inhibiting the occurrence of side reactions. Currently, organic materials (such as polyamide^[^ ^14^ ^]^ and polyacrylonitrile^[^ ^15^ ^]^) and inorganic materials (including porous carbon,^[^ ^16^ ^]^ TiO_2_,^[^ ^17^ ^]^ CaCO_3_,^[^ ^18^ ^]^ and ZrO_2_^[^ ^19^ ^]^) are utilized to establish protective interface layers on the zinc metal surface.

It is worth noting that the interface layer formed based on the traditional scraping process often has poor adhesion and excessive thickness. This uneven layer distribution is not conducive to ion migration kinetics. Atomic‐scale in situ modification technology has been used as a more effective and controllable anode interface protection method due to its advantages in thickness and uniformity. Zhang et al. in situ synthesized Zn@ZnPO insulating protective layer on zinc foil by a simple hydrothermal technique as an ion transport medium.^[^ ^20^ ^]^ The insulating Zn@ZnPO layer not only homogenizes the distribution of Zn^2+^ ions on the electrode surface but also effectively hinders the growth of dendrites and electrode corrosion. Yang et al. proposed the concept of “ion carrier” and used metal‐organic framework nanosheets as recyclable dynamic Zn^2+^ carriers to achieve reliable zinc deposition behavior.^[^ ^21^ ^]^ The self‐optimized zinc anode exhibits an exceptional morphology and deposition orientation, which significantly prolong the cycle life and leave a by‐product‐free surface. Zhu et al. developed an electrostatic field layer for assembling high‐speed and dendrite‐free zinc metal anodes.^[^ ^22^ ^]^ The Co(TAPC) with planar and large conjugated ring structure can be preferentially adsorbed on the metal zinc anode, which effectively inhibits the side reaction between zinc and water and balances the space electric field, resulting in high rate and dendrite‐free zinc deposition behavior. Zhang et al.used a fast room temperature wet chemical method to integrate a hydrophobic and fast conductive zinc hexacyanoferrate (HB‐ZnHCF) interphase layer on the zinc metal.^[^ ^23^ ^]^ This strategy effectively solves irreversible problems such as dendrite growth and corrosion of zinc anodes. The hydrophobic and dense HB‐ZnHCF interface phase effectively isolates the direct erosion of the Zn surface by water molecules, and the HB‐ZnHCF with excellent Zn^2+^ transfer coefficient can meet the rapid transport of Zn^2+^. Mi et al.designed a solid electrolyte interface rich in ZnF_2_ and Zn_3_N_2_ to stabilize the interfacial charge transfer kinetics of zinc anodes.^[^ ^24^ ^]^ Due to the electronegativity advantage of amino (‐NH_2_) in collagen, the Zn (002) crystal plane is preferentially exposed, and the charge transfer kinetics of the Zn anode are significantly optimized. The modified zinc anode obtained an impressive cumulative capacity of 7500 mAh cm^−2^ at 30 mA cm^−2^. The extensive research on the artificial interface layer still has great commercial application potential.^[^ ^25^ ^]^

In this paper, a ZnO‐SiO_2_ composite interface phase with a dredging induction effect is synthesized directly on the surface of zinc foil by a simple one‐step hydrothermal method. The interface phase has the dual protection effect of optimized wettability and electric field regulation function. The hydrophilic SiO_2_ layer can optimize the desolvation process of hydrated zinc ions to reduce the nucleation barrier of zinc ions. At the same time, the stable zinc oxide layer can adjust the electric field distribution on the zinc surface and induce uniform zinc nucleation behavior. The service life of the zinc symmetric battery based on Zn@ZSCP is more than 2500 h. In the potential range of 0.1–1.6 V, the zinc‐iodine full cell based on Zn@ZSCP anode and biomass‐derived porous carbon cathode showed excellent rate performance and long‐term cycle stability. This simple and reasonable structural design improves the practicability of aqueous zinc ion batteries to a certain extent and helps to develop more efficient and environmentally friendly zinc metal batteries.

## 2 Results and Discussion

### 2.1 Fabrication and Characterization of Zn@ZSCP Modified Anodes

**Figure 1:**
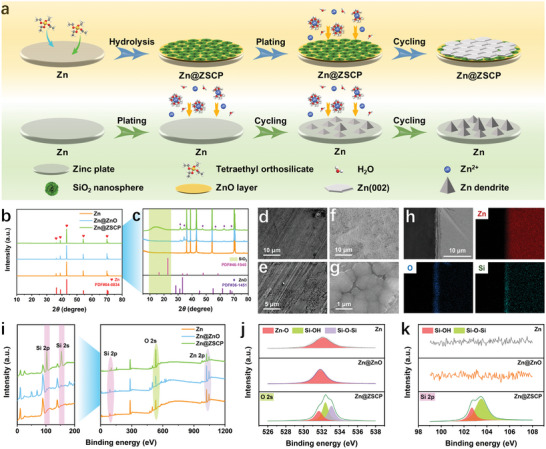
a) Schematic diagram of the fabrication process of Zn@ZSCP composite foil and inhibition of zinc dendrite. b) X‐ray diffraction (XRD) patterns of pure Zn, Zn@ZnO and Zn@ZSCP. Field‐emission scanning electron microscopy (FESEM) images of the c) pure Zn and d) Zn@ZSCP surface. e) X‐ray photoelectron spectroscopy (XPS) spectra of pure Zn, Zn@ZnO, and Zn@ZSCP as well as high resolution spectral f) Si 2p and g) O 2s XPS spectra.

The synthesis process of Zn@ZSCP and its inhibition effect on zinc dendrites are shown in **Figure** **1**a. In a high‐temperature confined space, the enhanced chemical activity of water will be more easily dissociated into hydrogen ions and hydroxide ions, which can accelerate the oxidation process of zinc. Benefiting from the reaction conditions of oxidants and protons provided by dissolved oxygen and water molecules, the surface of the zinc foil first reacts to form zinc‐based hydroxides (such as Zn(OH)_2_), which are then dehydrated to form ZnO. It is worth noting that studies have confirmed that alcohol‐aqueous solution can also accelerate the oxidation process of the whole reaction process.^[^ ^26^ ^]^ At the same time, the hydrolysis of tetraethyl orthosilicate forms a large number of hairy spherical SiO_2_. The hydrolysis and condensation reaction of ethyl orthosilicate can be expressed by the total reaction formula:^[^ ^27^ ^]^ Si(OCH_2_CH_3_)_4_ + 2H_2_O  = SiO_2_ + 4C_2_H_5_OH. Ethanol plays a catalytic role in the hydrolysis and condensation reactions of tetraethyl orthosilicate mainly by providing a solvent environment, participating in the stability of the reaction intermediates, and promoting the hydrolysis and condensation reactions by working with water. Finally, the ZSCP composite phase is in situ attached to the surface of the zinc foil. The XRD patterns of pure zinc foil, Zn@ZnO, and Zn@ZSCP all showed obvious characteristic peaks of Zn metal (Figure 1b). In addition, Zn@ZnO and Zn@ZSCP showed obvious zinc oxide characteristic peaks at high resolution (Figure 1c). A low‐intensity broad peak of Zn@ZSCP ≈15° corresponds to the presence of amorphous SiO_2_ nanospheres (Figures S1 and S2, Supporting Information).^[^ ^28^ ^]^ FESEM tests show that there are many bulges and depressions on the surface of commercial zinc foil in addition to polishing marks (Figure 1d,e), which is the root cause of the local dendrite intensification and uneven deposition of the zinc anode during the deposition process. Kinetics shows that the bulge is more conducive to the nucleation of zinc and the accumulation of dendrites, and ultimately leads to battery damage.^[^ ^29^ ^]^ Different from the bright color of the original zinc foil, the optical image shows that the surface of the Zn@ZSCP composite foil is dark black after modification (Figure S3, Supporting Information). The surface of Zn@ZSCP foil is covered by a layer of uniform and dense silica nanospheres with a diameter of ≈500–800 nm (Figure 1f,g). In contrast, the surface of Zn@ZnO without tetraethyl orthosilicate is relatively flat and attached with a large number of irregular fine particles, and there is no significant change in its color compared with that before modification (Figure S4, Supporting Information). The SiO_2_ and ZnO covered on the surface of Zn@ZSCP as a molecular sieve‐level multifunctional protective layer can adjust the electric field, which will be confirmed in subsequent tests. The cross‐section element distribution image of Zn@ZSCP foil further confirms the existence of the ZSCP interface protective layer, and the thickness of the protective layer is ≈1.5 µm (Figure 1h; Figure S5a, Supporting Information). The overall composition is SiO_2_ layer, ZnO layer, and pure Zn from the outside to the inside. The thickness of the SiO_2_ layer and ZnO layer is ≈1 and 0.5 µm, respectively. The chemical state of the metal foil is deeply revealed by XPS. The test results of Zn@ZSCP exhibit distinct signals at 103 and 154 cm^−1^, corresponding to Si 2p and Si 2s orbitals, respectively.^[^ ^30^ ^]^ Whereas no obvious Si signal was observed on pure Zn and Zn@ZnO, indicating the successful preparation of ZSCP interfacial phases (Figure 1i; Figure S5b, Supporting Information). The high‐resolution O 2s spectrum shows that Zn@ZnO has a stronger Zn‐O signal than pure Zn foil (Figure 1j), corresponding to more abundant zinc oxide. Additionally, the high‐resolution O 2s spectra of Zn@ZSCP reveal signals of Si─OH and Si─O─Si bonds.^[^ ^31^ ^]^ The two Si 2p signals at 102.7 and 103.6 cm^−1^ correspond to the characteristic stretching vibrations of the Si─OH and Si─O─Si bonds (Figure 1k), respectively,^[^ ^32^ ^]^ which is consistent with the high‐resolution O 2s spectra.

### 2.2 Electrochemical Performance of the Zn@ZSCP Modified Anodes

**Figure 2:**
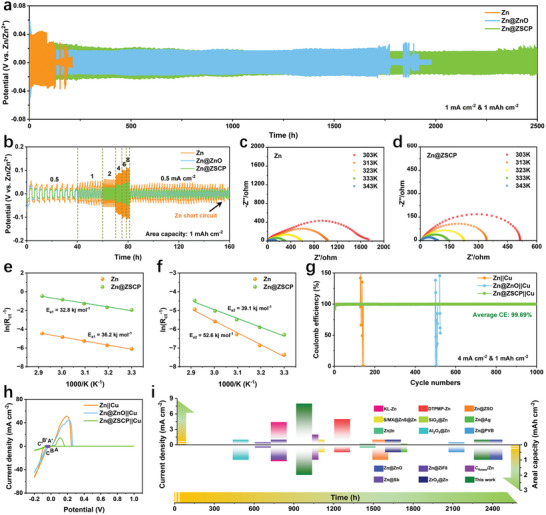
Electrochemical performance of the Zn, Zn@ZnO, and Zn@ZSCP symmetric cells. a) Galvanostatic voltage curves of the symmetrical battery at 1 mA cm^−2^ and 1 mAh cm^−2^. b) The rate performance. c,d) Nyquist electrochemical impedance spectroscopy (EIS) plots at different temperatures of 30–70 °C before cycling. e,f) Arrhenius curves and comparison of activation energies (Ea) of pure Zn and Zn@ZSCP electrodes. g) Coulombic efficiency (CE) at 4 mA cm^−2^ and 1 mAh cm^−2^ of pure Zn//Cu, Zn@ZnO//Cu and Zn@ZSCP//Cu asymmetrical cells. h) CV curves of Zn plating/stripping of asymmetrical cells at 0.1 mV s^−1^. i) The comparison of cyclic reversibility between this work and previous reports.^[^ ^12^, ^19^, ^28^, ^33^, ^34^ ^]^

To verify the effectiveness of the ZSCP layer in protecting the zinc anode, zinc symmetric cells were tested at different surface current densities. Compared with pure Zn, the Zn@ZSCP electrode exhibits lower voltage fluctuation and nucleation potential (**Figure** **2**a; Figures S6 and S7, Supporting Information), indicating a more reversible zinc deposition/stripping process with a smaller nucleation driving force.^[^ ^35^ ^]^ In addition, the Zn@ZSCP symmetrical battery not only has a more stable voltage curve but also exhibits a lower voltage fluctuation. For example, the stable nucleation potential of the Zn@ZSCP electrode at 0.5 mA cm^−2^ is only 32 mV, while the pure zinc is as high as 70 mV. The short circuit or fault of a pure zinc symmetrical battery after 100 cycles is accompanied by an obvious voltage jump. Impressively, the Zn@ZSCP symmetric cell can maintain stable output for up to 2500 h at 1 mA cm^−2^, far exceeding Zn@ZnO (1700 h) and pure zinc (120 h). Even at high current densities of 4 and 8 mA cm^−2^, Zn@ZSCP can still maintain a stable state for more than 2800 and 2400 cycles. The Zn@ZSCP symmetric cell always maintains a smaller nucleation potential and a smoother voltage curve in the range of regional current density (Figure 2b; Figure S8, Supporting Information). This significantly extended service life is mainly attributed to the dual protection mechanism provided by the zinc oxide layer and the silica layer, which is more helpful in alleviating the mechanical stability damage caused by the volume change of the electrode during the long‐term cycle. The electroplating/stripping behavior of the Zn@ZSCP symmetric battery was studied by rate experiment and Nyquist curve. Although the contact resistance between the modified zinc metal anode and the electrolyte is slightly increased due to the presence of the insulating interface phase (Figures S9 and S10, Supporting Information), this does not affect the excellent charge transfer kinetics of Zn@ZSCP. In addition, the measured activation energy (Ea) of the electrode was evaluated for the desolvation barrier of Zn^2+^ by calibrating the Nyquist EIS diagram at different temperatures of 10–50 °C before cycling (Figure 2c,d). According to the Arrhenius equation, the decrease of the Ea value of the Zn@ZSCP electrode corresponds to the accelerated desolvation (Figure 2e,f), which is beneficial to the improvement of ion transfer kinetics.

The thermodynamic ability of zinc to form temporary clusters on the substrate was compared by constructing Zn//Cu asymmetric cells. A lower nucleation potential corresponds to a more uniform and orderly nucleation process.^[^ ^36^ ^]^ At 0.5 and 1 mA cm^−2^, Zn@ZSCP has a significantly lower nucleation potential and better charge‐discharge efficiency (Figures S11 and S12, Supporting Information). Even at a high current density of 4 mA cm^−2^, the Zn@ZSCP (53 mV) is still lower than that of pure zinc (135 mV) (Figures S13 and S14, Supporting Information), indicating that the ZSCP layer has a positive uniform induction effect on zinc nucleation. In addition, the average coulombic efficiency of the Zn@ZSCP//Cu asymmetric cell is close to 100% (99.69%) in more than 1000 cycles (Figure 2g), further revealing excellent cycle stability. Cyclic voltammetry (CV) measurements of Zn@ZSCP//Cu asymmetric cells were performed at a scan rate of 0.5 mV s^−1^ and a potential range of −0.2–1 V (Figure 2h; Figure S15, Supporting Information). The potential difference between the curve intersection and the initial dissolution of zinc is usually used to determine the nucleation potential of zinc.^[^ ^37^ ^]^ The nucleation potential of Zn@ZSCP is 15 mV, which is significantly lower than that of pure Zn (39 mV), indicating that Zn@ZSCP has lower nucleation barriers and faster kinetics (Figure S15, Supporting Information). Overall, the Zn@ZSCP||Zn@ZSCP symmetric cell results exceed many previous reports (Figure 2i).

### 2.3 The Effect on Hydrogen Evolution and Corrosion by the Modified Anodes

**Figure 3:**
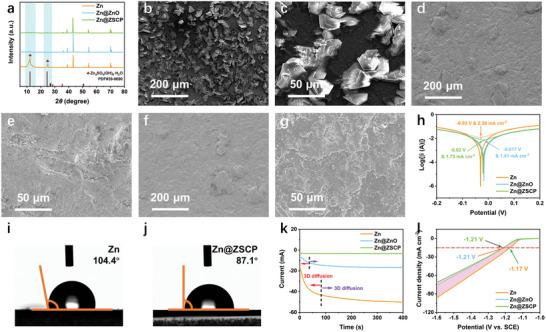
a) The XRD pattern of the electrodes immersed in 2 M ZnSO_4_ aqueous solution electrolyte after 10 days. The FESEM images of the electrode after immersion, b,c) pure Zn, d,e) Zn@ZnO, and f,g) Zn@ZSCP. h) Linear polarization curves for describing corrosion of Zn anodes. Contact angle test of i) pure Zn and j) Zn@ZSCP composite foil. k) Chronoamperometry curves of various electrodes at an overpotential of −150 mV. l) Linear polarization curves of electrodes in 1 m ZnSO_4_ aqueous electrolyte.

The corrosion resistance of the modified zinc anode was further demonstrated by immersing the electrode in 2 m ZnSO_4_ electrolyte. The XRD results showed that an obvious characteristic peak of alkaline zinc sulfate by‐products was formed on the pure zinc electrode after 10 days of immersion, while Zn@ZnO and Zn@ZSCP only showed a small peak intensity of by‐products (**Figure** **3**a). The FESEM images are consistent with the XRD results. The surface of pure zinc is severely damaged and covers a large area of alkaline zinc sulfate (ZnSO_4_(OH)_6_·H_2_O) by‐products (Figure 3b,c).^[^ ^38^ ^]^ Fortunately, Zn@ZnO and Zn@ZSCP maintain a relatively flat surface structure without obvious by‐products (Figure 3d–g). The side reaction of the Zn anode in the ZnSO4 electrolyte can be expressed as follows:

(1) $$\mathrm{Zn}\to {\mathrm{Zn}}^{2+}+2e^{-}$$

(2) $$H_{2}O+2e^{-}↔2OH^{-}+H_{2}$$

(3) $$Zn^{2+}+OH^{-}+{\mathrm{SO}}_{4}^{2-}+xH_{2}O↔Zn_{4}SO_{4}{\left(\mathrm{OH}\right)}_{6}\cdot xH_{2}O$$

The corrosion inhibition mechanism of the electrode was deeply analyzed by linear polarization curves (Figure 3h). In 2 m ZnSO_4_ electrolyte, Zn@ZnO and Zn@ZSCP showed significantly enhanced corrosion inhibition effects. The calculated results show that the corrosion current density (1.73 mA cm^−2^) and corrosion potential (−0.02 V) of Zn@ZSCP are lower than those of pure zinc (2.38 mA cm^−2^ & −0.03 V). The more positive corrosion potentials and smaller corrosion current density indicate that the Zn@ZSCP anode has stronger inert corrosion activity. The wettability of electrolytes plays an important role in the deposition and/or stripping process of zinc anode. A smaller contact angle means better surface wettability, which facilitates the rapid diffusion of Zn^2+^ on the anode surface.^[^ ^39^ ^]^ The contact angle of the Zn@ZSCP anode (87.1°) is significantly smaller than that of pure zinc (104.4°) (Figure 3i,j), which contributes to the accelerated penetration of the electrolyte. Similar results were obtained for Zn@ZnO anode (Figure S16, Supporting Information). Nevertheless, the presence of the ZSCP interface phase hinders the direct contact between the electrolyte and the zinc foil, thereby inhibiting the corrosion of the zinc foil.

Chronoamperometry curves are a crucial tool for examining the morphology of zinc deposition on the anode surface. The zinc deposition behavior of the anode can be further analyzed by the change of the polarization current. In general, zinc ion deposition can be divided into 2D and 3D deposition processes.^[^ ^40^ ^]^ In the 2D deposition process, zinc ions will preferentially adsorb on the irregular surface which is easy to deposit, thus aggravating the growth of dendrites. In the 3D deposition process, zinc ions are induced to perform uniform deposition behavior, thereby reducing the trend of zinc dendrite growth.^[^ ^41^ ^]^ Therefore, a short 2D diffusion process is conducive to stable long‐period zinc deposition/stripping. As shown in Figure 3k, at a constant potential of −150 mV, the polarization current of pure zinc decreases rapidly in the first 90 s and then gradually decreases and tends to be stable in ≈300 s. The polarization current of Zn@ZSCP and Zn@ZnO anodes tends to be stable after 50 s and 3 s, respectively, showing excellent 3D deposition behavior, which is the main reason for maintaining long‐cycle stability. As a common electrolyte for aqueous zinc ion batteries, ZnSO_4_ aqueous solution is usually weakly acidic, which makes the zinc anode easy to corrode and release hydrogen during continuous deposition/stripping. Electrochemical corrosion and self‐corrosion caused by direct contact between electrolyte and electrode surface during electrode operation are inevitable. Therefore, it is very important to minimize anode corrosion by using an interface regulation strategy. Linear sweep voltammetry (LSV) can be used to evaluate the hydrogen evolution reaction (HER) inhibition effect of the electrode.^[^ ^42^ ^]^ The current density of different electrodes based on hydrogen evolution reaction in 2 m ZnSO_4_ aqueous electrolyte was tested at a scan rate of 5 mV s^−1^ (Figure 3l). Zn@ZSCP effectively inhibited the electrochemical water decomposition on the surface of the zinc anode. The water splitting potential shifted from −1.17 V for pure zinc to −1.21 V for Zn@ZSCP and Zn@ZnO at a current density of 15 mA cm^−2^. The results show that Zn@ZSCP can reduce the activity of water and prevent the occurrence of harmful interfacial side reactions.

### 2.4 The Zn Plating Mechanism and the Effect on Repeated Plating/Stripping of Modified Anodes

To further reveal the deposition mechanism of the electrode, the XRD patterns and FESEM images of the electrode after 1 and 10 cycles at 1 mA cm^−2^ and 1 mAh cm^−2^ were compared (Figures S17 and S18, Supporting Information). For pure zinc electrode, the characteristic peak intensity of the (101) crystal plane ≈43° decreased significantly after 10 cycles, and the characteristic peak intensity of the (002) crystal plane ≈36° and (103) and (110) crystal planes ≈70° increased significantly, accompanied by the formation of obvious by‐product ZnSO_4_(OH)_6_·xH_2_O. This non‐preferential deposition behavior of zinc corresponds to the irregular zinc deposition process, and the tip effect and the lack of nucleation sites are the main reasons for this phenomenon.^[^ ^43^ ^]^ The cumulative effect will promote the further growth of dendrites and aggravate the production of various by‐products. Compared with pure Zn, Zn@ZSCP has a significant enhancement on the (002) crystal plane after 10 cycles, indicating that the (002) crystal plane is the preferred deposition crystal plane of Zn@ZSCP anode. The FESEM images of the surface of pure zinc and Zn@ZSCP after cycling showed similar effects. There are many disordered zinc dendrites on the surface of the pure zinc electrode after 10 cycles, while the surface of Zn@ZSCP is still smooth after 100 cycles. Although the XRD spectrum of the Zn@ZSCP anode after 100 cycles shows a slight side reaction on the electrode surface, the overall electrode still retains obvious surface characteristics, indicating that the ZSCP interface phase has a solid surface protection effect. The Zn@ZnO electrode also exhibits an enhanced dendrite suppression effect (Figure S19, Supporting Information).

**Figure 4:**
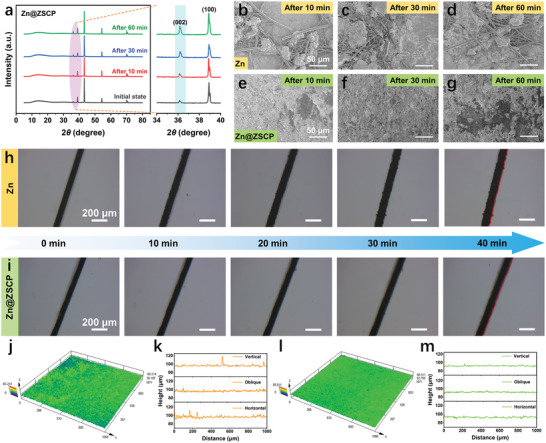
a) The XRD patterns of Zn@ZSCP electrode after deposition at 2 mA cm^−2^ for different time periods. FESEM images of b–d) pure Zn and e–g) Zn@ZSCP after deposition at 2 mA cm^−2^ for different time periods. In situ optical observation of h) pure Zn and i) Zn@ZSCP at 5 mA cm^−2^. The 3D images of the confocal laser microscope (CLSM) and the corresponding line roughness of j,k) Pure Zn and (l,m) Zn@ZSCP after 100 cycles.

The same conclusion was further confirmed by the continuous deposition of the electrode at a surface current density of 2 mA cm^−2^. The results show that the strength of the (002) crystal plane of the Zn@ZSCP electrode increases with the deposition process (**Figure** **4**a). It is worth noting that obvious disordered zinc dendrites appeared on the surface of pure zinc after continuous deposition for 60 min (Figure 4b–d). The Zn@ZSCP anode maintained a smooth surface after 60 min (Figure 4e–g), which can be attributed to the good affinity of the ZSCP protective layer to zinc.^[^ ^44^ ^]^ The surface deposition process based on an in situ optical microscope shows the same conclusion. At a current density of 5 mA cm^−2^, with the increase of the amount of deposited zinc, the surface of the pure Zn electrode showed an obvious burr phenomenon at 30 min. The Zn@ZSCP electrode still maintains a flat surface after 40 min of continuous zinc deposition. The surface morphology of symmetrical zinc batteries after different cycles was characterized by CLMS, which further confirmed the different deposition behaviors of Pure Zn and Zn@ZSCP symmetrical zinc batteries. After 100 cycles, severe corrosion and dendrites appeared on the surface of bare Zn, with significantly higher roughness (Figure 4j,k). However, the Zn@ZSCP surface exhibits a relatively smooth and flat surface after 100 cycles (Figure 4l,m). This is consistent with the previous SEM images and in situ optics and reflects the advantages of the ZSCP interface phase in the homogenization of Zn^2+^ flux. In summary, the ZSCP protective layer has a significant gain effect on inducing uniform zinc deposition.

### 2.5 Optimized Deposition Mechanism of Modified Anodes based on Density Functional Theory (DFT) Calculation

**Figure 5:**
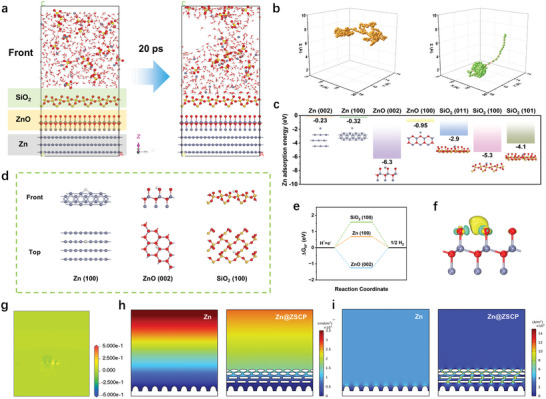
a) MD simulation snapshot (front view) of Zn^2+^ ions and H_2_O molecule on the surface of Zn@ZSCP composite foil in 2 m ZnSO_4_ aqueous electrolyte. b) The migration trajectories of Zn^2+^ near Zn foil (left) and Zn@ZSCP (right) composite foil. c) The adsorption of Zn^2+^ on Zn and SiO_2_. d) The adsorption sites and e) the corresponding hydrogen adsorption Gibbs free energy (ΔG_H*_) for HER in Zn (100), ZnO (002), and SiO_2_ (100) lattices. f) The charge distribution and g) the corresponding slice of the electron density difference map at the ZnO interphase (front view). Note: Yellow represents electron‐gain behavior, and blue represents electron‐loss behavior. f) Simulated Zn^2+^ ion field of Zn and Zn@ZSCP surfaces. g) Simulated electrolyte current density field of Zn and Zn@ZSCP surfaces.

The interaction of Zn atoms and H_2_O molecules with Zn metal and ZSCP protective layer was further studied by using the Density Functional Theory (DFT) method. Molecular dynamics (MD) simulation was used to further reveal the dynamic behavior of Zn^2+^ ions in 2 m ZnSO_4_ aqueous electrolyte. As shown in **Figures** **5**a and S20 (Supporting Information), the molecular trajectories of Zn^2+^ in 2 m ZnSO_4_ electrolyte near the ZSCP interface are simulated. Part of Zn^2+^ rapidly migrated to the ZSCP interface after 5 ps, while the other part of Zn^2+^ further approached the ZSCP interface. It is worth noting that there is little accumulation of SO_4_^2−^ anions near the ZSCP interface, which can reduce the interference with the behavior of Zn^2+^ deposition. Although a large amount of water molecules is adsorbed near the ZSCP interface, the ZSCP interface phase can firmly protect the zinc anode from the erosion of the electrolyte. For the pure Zn electrode, the movement of Zn^2+^ and SO_4_^2−^ ions did not show obvious rules, and the overall movement was disordered (Figure S21, Supporting Information). This not only confirms the hydrophilicity of SiO_2_ but also illustrates the ability of the ZSCP interface to regulate the electric field of Zn^2+^. The migration trajectory of zinc ions near the Zn and ZSCP interfaces further confirms this conclusion (Figure 5b). The Zn^2+^ above the pure Zn interface exhibits a disordered Brownian motion, while the Zn^2+^ above the ZSCP rapidly migrates to the vicinity of the interface, indicating that the ZSCP interface has a good adsorption capacity for Zn^2+^, which contributes to the rapid replenishment of Zn^2+^ during the deposition process.

Detailed adsorption calculation results show that both ZnO and SiO_2_ surfaces show stronger adsorption for Zn^2+^ and H_2_O molecules (Figure 5c; Figure S22, Supporting Information). The strong adsorption of Zn^2+^ ion molecules is beneficial in reducing the energy loss of zinc ion diffusion during zinc deposition (Figures S23–S25, Supporting Information). The strong adsorption of H_2_O molecules not only contributes to the penetration of the electrolyte but also helps to accelerate the desolvation process of hydrated zinc ions (Figures S26–S28, Supporting Information). It is worth noting that the calculation results show that the ZSCP interface can effectively alleviate the HER on the electrode surface (Figure 5d,e; Figures S29–S31, Supporting Information). Taking the most easily affected Zn (100), ZnO (002), and SiO_2_ (100) crystal planes as examples, the ZnO (002) and SiO_2_ (100) crystal planes still have higher ΔG_H*_ values (Figures S32–S34, Supporting Information), which means that the hydrogen evolution reaction based on this crystal plane requires more energy, which is conducive to inhibiting the HER of the electrode.^[^ ^45^ ^]^ In addition, oxygen atoms with different charges help to homogenize the charge distribution around Zn^2+^. Therefore, the existence of the ZnO and SiO_2_ interface layer helps to adjust the electric field distribution on the anode surface and promotes the uniform deposition of zinc ions (Figure 5f,g). ZnO and SiO_2_ can contribute some electrons to Zn^2+^ to reduce the charge density in the local area (Figures S35 and S36, Supporting Information), which is crucial for the long‐term stable cycle of the zinc anode.

The effect of the ZSCP interface on the deposition behavior of Zn^2+^ ions on the electrode surface was further studied by COMSOL multi‐physics software. The vertical ellipse at the bottom represents the uneven Zn surface, and the elliptical and rectangular distributions at the top correspond to the interface phases of SiO_2_ and ZnO, respectively. The simulation results show that there is a significant Zn^2+^ ion concentration gradient above the bare zinc anode (Figure 5h), which is not conducive to stable zinc deposition behavior. At the same time, the protruding surface of the bare zinc anode will gradually accumulate a large electrolyte current density, which will lead to uncontrolled continuous deposition of the zinc tip during the plating process and cause obvious zinc dendrites. On the other hand, the zinc anode modified by the ZSCP interface phase shows a more uniform Zn^2+^ ion concentration distribution (Figure 5i), and the prominent zinc surface shows a more stable current density. In summary, the ZSCP interface is very effective in combing the zinc deposition behavior on the zinc anode surface. These comprehensive advantages promote the excellent comprehensive performance of the Zn@ZSCP anode.

### 2.6 The Full‐Cell Performances of the Modified Anodes

**Figure 6:**
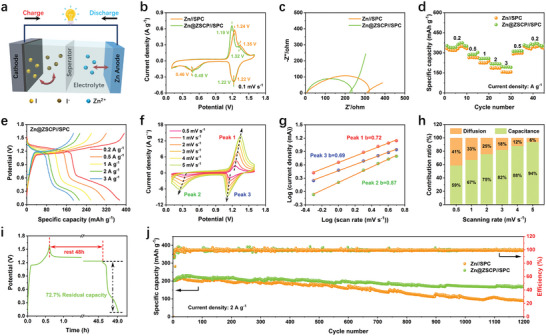
a) Working mechanism of the Zn//SPC and Zn@ZSCP//SPC full cells. b) The second cycle CV curves. c) EIS curves before cycling and (c) slopes of impedance and low frequency. d) Rate performance. e) GCD curves of Zn@ZSCP//SPC. f,g) Relationship of log (peak current) and log (scan rate) (The relationship between current (i) and sweep rate (v) can be determined by *i = av^b^* and *log(i) = log(a)+b log(v)*) of Zn@ZSCP//SPC. i) The self‐discharge test of Zn@ZSCP//SPC. j) long‐term cycling stability of the full cells.

To evaluate the practical application of the Zn@ZSCP anode, we conducted tests using a zinc‐iodine cell with an SPC cathode (**Figure** **6**a). ZnI_2_ is used as an iodine source, and the electrochemical performance of the Zn//SPC and Zn@ZSCP//SPC full cells was compared with that of bare Zn anodes.^[^ ^46^ ^]^ The XRD pattern of SPC is shown in Figure S37 (Supporting Information), the amorphous peak at 20° corresponds to the (002) peak of carbon, and its morphology appears as an irregular block structure (Figure S38, Supporting Information). The CV curves of the full cells are compared in Figure 6b. Both the Zn//SPC and Zn@ZSCP//SPC cells exhibited similar redox peaks, indicating that the protective coating did not affect the reversible intercalation chemistry of the battery.^[^ ^47^ ^]^ In the 2 m ZnSO_4_ + 0.2 m ZnI_2_ electrolyte, the CV curves show a reduction peak at 1.22 V and an oxidation peak at 1.35 V, indicating that the introduction of I^−^ into the electrolyte will lead to the generation of I_3_^−^ during the charge process, thus form a reversible I_3_^−^/I^−^ redox process for the electrochemical energy storage. While a pair of redox peaks ≈0.46 and 1.24 V corresponded to the reversible conversion reaction of S/ZnS.^[^ ^48^ ^]^ The CV curves revealed that the Zn@ZSCP//SPC cell exhibited a higher current density compared to the Zn//SPC cell, suggesting enhanced capacity and electrochemical activity. Moreover, the Zn@ZSCP//SPC cell showed smaller polarization potential and higher exchange current density than the pure Zn, indicating better redox kinetics. The Nyquist curve displayed lower solution resistance and charge transfer resistance for the Zn@ZSCP//SPC cell compared to the Zn//SPC cell (Figure 6c). The resistance values were measured at 2.3 and 118 Ω respectively, which were lower than the Zn//SPC cell (3 and 167 Ω), indicating faster electron mobility.^[^ ^49^ ^]^ Importantly, the smaller slope of the Nyquist curve in the low‐frequency region for the Zn@ZSCP//SPC battery indicated better Zn^2+^ ion diffusion kinetics (Figure S39, Supporting Information).

The multiplicative capacity of the cells was tested at various current densities (Figure 6d,e; Figure S40, Supporting Information). The Zn@ZSCP//SPC cell demonstrated improved discharge capacity compared to the Zn//SPC cell at subsequent current densities. This improvement can be attributed to the lower charge transfer resistance of the Zn@ZSCP//SPC cells. Notably, when the current density returned to 0.2 A g^−1^, the Zn@ZSCP//SPC cell maintained a significantly higher specific capacity of 374.1 mAh g^−1^, while the Zn//SPC cell experienced a substantial decline in capacity (348.7 mAh g^−1^). Even at a high current density of 3 A g^−1^, the Zn@ZSCP//SPC full cell still retains an excellent rate performance of 195.7 mAh g^−1^. By exploring the peak current and scanning rate in the CV curve of the battery (Figure 6f–h; Figure S41, Supporting Information), it can be seen that the energy storage process based on the sulfurized porous carbon battery is jointly dominated by capacitance contribution and diffusion contribution. As the scan rate increases, the capacitance contribution of the battery gradually dominates, which helps the battery to provide excellent electrochemical kinetics at high rates. This is the main reason why the battery maintains excellent rate performance at high current density. The self‐discharge behavior of the cells was monitored to assess the interfacial stability of the cells. The cells were charged to 1.6 V, rested for 48 h, and then discharged to 0.1 V (Figure 6i; Figure S42, Supporting Information). The Zn@ZSCP//SPC cell exhibited a higher open circuit potential and retained more residual specific capacity up to 72.7% (higher than 65.9% of pure Zn), indicating its superior ability to suppress self‐discharge.^[^ ^34^ ^]^

The long‐term cycling performance of both cells was further investigated at 0.2 and 2 A g^−1^ (Figure 6j; Figure S43, Supporting Information). The Zn@ZSCP//SPC battery still maintains more than 90% of the residual capacity output after 100 cycles at a current density of 0.2 A g^−1^ and has been superior to Zn//SPC during this period. Even under a high current density of 2 A g^−1^, the Zn@ZSCP//SPC cells demonstrated significantly enhanced residual discharge specific capacity after 1200 cycles. The rapid capacity decay observed in the pure Zn anode may be attributed to uncontrolled dendrite growth and continuous corrosion.^[^ ^34^ ^]^ These results indicate that the ZSCP interfacial phase effectively mitigates harsh Zn dendrites and prolongs the long‐term cycling stability of batteries.

## 3 Conclusion

In this study, a new ZSCP interface phase with a combing effect was synthesized directly on zinc foil by a simple hydrothermal method. The SiO_2_ and ZnO layers with strong adsorption effect on H2O molecules can not only accelerate the de‐dissolution process of hydrated zinc ions during zinc deposition to reduce the zinc nucleation barrier but also regulate the charge density and electric field distribution of deposited Zn^2+^ by stable SiO_2_ and ZnO, which can induce ordered zinc deposition behavior. Based on this innovative ZSCP interface phase, Zn@ZSCP symmetric batteries have an excellent service life. The novel zinc‐iodine battery based on Zn@ZSCP anode and low‐cost biomass sulfurized porous carbon cathode also exhibits excellent rate performance and good long‐term stability. This simple and reasonable structural design improves the practicability of aqueous zinc ion batteries to a certain extent and helps to develop more efficient and environmentally friendly sustainable zinc metal batteries.

## Conflict of Interest

The authors declare no conflict of interest.

## Author Contributions

D.F.G. conceived the project and designed the experiments. D.F.G. and F.Y.L. contributed to sample preparation and experiments. D.F.G. carried out the theoretical calculation and data collation and completed the first draft writing and form analysis. B.Z. participated in the manuscript examination. All authors discussed the results and commented on the manuscript.

## Supporting information



Supporting Information

## Data Availability

The data that support the findings of this study are available from the corresponding author upon reasonable request.
