# Supplementary material for: The ZnO‐SiO2 Composite Phase with Dual Regulation Function Enables Uniform Zn2+ Flux and Fast Zinc Deposition Kinetics Toward Zinc Metal Batteries
Source: Adv Sci (Weinh). 2024 Dec 4;12(4):2411995. doi: 10.1002/advs.202411995 (PMC11775536; doi:10.1002/advs.202411995)
Supplement: Supplementary file 1 — Supporting Information [file ADVS-12-2411995-s001.docx]

**Supporting Information**

The ZnO-SiO_2_ Composite Phase with Dual Regulation Function Enables Uniform Zn^2+^ Flux and Fast Zinc Deposition Kinetics Toward Zinc Metal Batteries

*Dongfang Guo**^1,2^, Fengyu Li^1,2^, Bin Zhang^1,2,*^*

^1^ School of Physics and Microelectronics, Zhengzhou University, Zhengzhou, 450001, China

^2^ School of Physics and Laboratory of Zhongyuan Light, Zhengzhou University, Zhengzhou, 450001, China

E-mail: [1184669315@qq.com](mailto:1184669315@qq.com) (D.F. G.); [fengyuli_zzu@163.com](mailto:fengyuli_zzu@163.com) (F.Y. L.); [zb1967@zzu.edu.cn](mailto:zb1967@zzu.edu.cn) (B. Z.)

**Experimental section**

*Preparation of Zn@ZSCP and Zn@ZnO anode.* 5 mL tetraethyl orthosilicate (TEOS) was dropped into 40 mL absolute ethanol and stirred for 30 min, then 120mL deionized water was added to continue stirring for 30 min. The mixed solution and a 100 μm thick zinc foil (4.5 cm × 15 cm) were transferred to a 200 mL Teflon-lined stainless steel autoclave at 120℃ for 36 h. After that, the metal foil was taken out, cleaned with deionized water. The final product obtained by the treatment is the Zn@ZSCP anode, which is cut into a suitable size (Φ = 12 mm) for testing. It is worth noting that all modified zinc foils need to be polished before use to remove the protective layer on the other side to reduce the impact on the overall experiment, in which one side with a protective layer is in direct contact with the glass fiber diaphragm. The synthesis step of Zn@ZnO anode is consistent with that of Zn@ZSCP. In contrast, the Zn@ZnO anode does not add TEOS during the preparation process.

*Preparation of SiO_2_ Nanospheres.* 30.0 mL of absolute ethanol, 5.0 mL of deionized water and 2.0 mL of ammonia solution were mixed and stirred in a 100 mL beaker. Then, 0.2 mL tetraethyl orthosilicate was slowly added to the mixed solution and stirred at room temperature for 30 min. Afterwards, 3.8 mL TEOS was added to the solution and the reaction was continued at room temperature for 12 h. The reaction solution was centrifuged at 6000 rpm for 10 min to separate and collect SiO_2_ nanospheres, washed repeatedly with deionized water and ethanol, and dried in air to obtain the final product.

*Preparation of sulfurized porous carbon (SPC) cathode.* Peanut shells were carbonized at 500℃ for 2 h in N_2_ atmosphere, and then immersed in 6M KOH solution for 12 h. The carbonized product was activated at 700℃ for 3 h and washed with dilute hydrochloric acid to remove excess KOH. After vacuum drying at 60℃ for 12 h, the initial porous carbon was obtained. After that, the porous carbon and the sublimed sulfur were uniformly mixed at a mass ratio of 1:1, and then the final sulfurized porous carbon was obtained in a tube furnace at 250℃ for 6 h under the protection of N_2_ atmosphere.

*The assembly of symmetric cells and asymmetrical cells.* Symmetric cells were assembled into CR2032-type coin cells in an air atmosphere, glass-fiber filter (GF-D, Whatman), metal anodes (Zn, Zn@ZnO and Zn@ZSCP) and 2M ZnSO_4_ aqueous solution were used as the separator, electrode and electrolyte, respectively. Asymmetrical cells were fabricated using metal anodes and copper foil as working and counter electrodes, respectively, where the diaphragm and electrolyte were the same as in the symmetric cells.

*The assembly of full Cell.* The full cell was assembled directly in air as a CR2032 button cell. Glass-fiber filter (GF-D, Whatman), metal anodes (Zn, Zn@ZnO and Zn@ZSCP) and a conventional aqueous solution (2 M ZnSO_4_ + 0.2 M ZnI_2_) were used as the separator, anode and electrolyte, respectively. Typically, SPC, carbon black and polyvinylidene fluoride (PVDF) were mixed in N-methyl-2-pyrrolidone solvent at a weight ratio of 8:1:1 to obtain a slurry and uniformly coated on a stainless steel mesh and dried at 60℃ for 12 h as the cathode of the full cell. The electrode plate was cut into a diameter of 12 mm after drying, and the mass of a single electrode plate was about 1.5-2 mg cm^-2^.

*Material Characterization.* Powder X-ray diffraction patterns were conducted by the D8 ADVANCE diffractometer using Cu Kα-radiation (λ= 1.5418). The micromorphology of the samples was measured by field-emission scanning electron microscopy (FESEM, JSM-7001F) and high-resolution transmission electron microscopy (HRTEM, JSM-2100). The surface element distribution was explored by energy dispersive X-ray spectroscopy. The surface chemical compositions were analyzed by X-ray photoelectron spectroscopy (XPS, ESCALAB 250Xi). Focus Laser Scanning Microscope images were tested based on Japan-Olympus-OLS4100. Electrochemical testing was performed on an electrochemical workstation CHI660E (Shanghai, China) and LANHE CT3001A (Wuhan, China).

*Electrochemical Performance Evaluation.* Galvanostatic charge/discharge curves, rate performance, and long-term cycling tests of the full cells at different current densities at room temperature were recorded on a LANHE CT3001A battery tester (Wuhan, China). The Electrochemical Impedance Spectroscopy (EIS) spectra (from 100 kHz to 0.01 Hz), Cyclic voltammetry (CV), Linear polarization curves and chronoamperometry (CA) were tested on CHI660E electrochemical workstation (Shanghai, China). The three-electrode system consists of a bare Zn or surface-modified Zn foil as the working electrode, a platinum foil as the counter electrode, and an Ag/AgCl electrode as the reference electrode. The CA tests were carried out in the three-electrode system at an overpotential of -150 mV. LSV was tested in the voltage range from -1.6 to -1 V at a scan rate of 5 mV s^-1^.

The EIS of symmetrical cells at different temperatures was measured. The linear relationship between charge transfer resistance (*R_ct_*) and temperature can be obtained by fitting the Arrhenius equation (1) ^[1]^:

$\frac{1}{R_{ct}}=Aexp(\frac{-E_{a}}{RT})$ *(1)*

Where *R_ct_* is the charge transfer resistance, and *A*, *T*, *R* and *E_a_* are constants, Kelvin temperature, ideal gas constant and activation energy, respectively.

*Calculation methods.* The whole density functional theory DFT calculation process is carried out by Material Studio, using the projector augmented wave potentials with a plane wave cutoff of 500 eV. The Perdew-Burke-Ernzerhof (PBE) functional within the generalized gradient approximation (GGA) were applied to describe ionic cores and exchange-correlation effects, respectively. The vacuum gap between periodic images was set to 15 Å to avoid interaction. The SCF tolerance were set to 10^-6^ eV/atom, respectively.

The surfaces were built to investigate the adsorption energies. The adsorption energies of the Zn^2+^ ions and H_2_O molecule on the Zn, ZnO and SiO_2_ surfaces were calculated as follows: *E_ad_* = *E_t_* - *E_s_* - *E^*^*, where *E_t_*, *E_s_*, *E^*^* were the total energy of Zn^2+^ ions and H_2_O molecule adsorbed on the surface, the surface energy, and the energy of individual Zn atom, respectively. The charge density changes of the Zn adsorbed on the surface were calculated following the equation (2):

$\rho=\rho_{t}-\rho_{s}-\rho^{*}$ *(2)*

where *ρ_t_*, *ρ_s_*, *ρ^*^* were the charge densities of Zn^2+^ adsorbed surfaces, individual the surfaces and Zn^2+^, respectively. When the above convergence criteria were satisfied, the structure optimization was completed.

The free energy profile for the HER was obtained based on the computational hydrogen electrode (CHE) model proposed by Nørskov and co-workers ^[2]^. In this model, the free energy of proton-electron pair is equal to that of 1/2 H_2_(g). The free energy change for each fundamental step was determined by equation (3):

$\Delta G=\Delta E+E_{ZPE}+T\Delta S$ *(3)*

where *∆E* is the difference of electronic energy directly obtained from DFT simulation. *E_ZPE_* is the variation of zero-point energy (ZPE), *∆S* is the entropy (S) change, *T* is the temperature (*T* = 298.15K) The *ZPE* and *S* were obtained by the vibrational frequencies. For the molecular in gas phase, H_2_ (g), the *ZPE* and *S* were taken from NIST database.

The current density distribution (also known as Zn^2+^ flux distribution) and the electric field distribution of Zn@ZSCP and pure Zn samples were numerically simulated by using "Cubic current distribution" in COSMOL Multiphysics. In the simplified two-dimensional model, the interlaced nanosheet structure was used to represent the ZSCP interface. The size of the entire two-dimensional model for the current density distribution analysis is set to 8 μm × 8 μm. The top of the geometry represents the cathode, the anode is at the bottom, and the vertical wall is assumed to be the insulation boundary. The distance between the top and bottom electrodes is 7 μm. The zinc deposit is elliptic. The electrode surface reaction is given by Butler-Volmer kinetic expression. The simulation parameters are as follows: the ion diffusion coefficient of ZnSO_4_ electrolyte is set to 2 × 10^-9^ m^2^ s^-1^, the initial concentration of Zn^2+^ is set to 2 mol L^-1^.

**Supplementary Figures**





**Fig. S1** XRD patterns of SiO_2_ nanospheres.


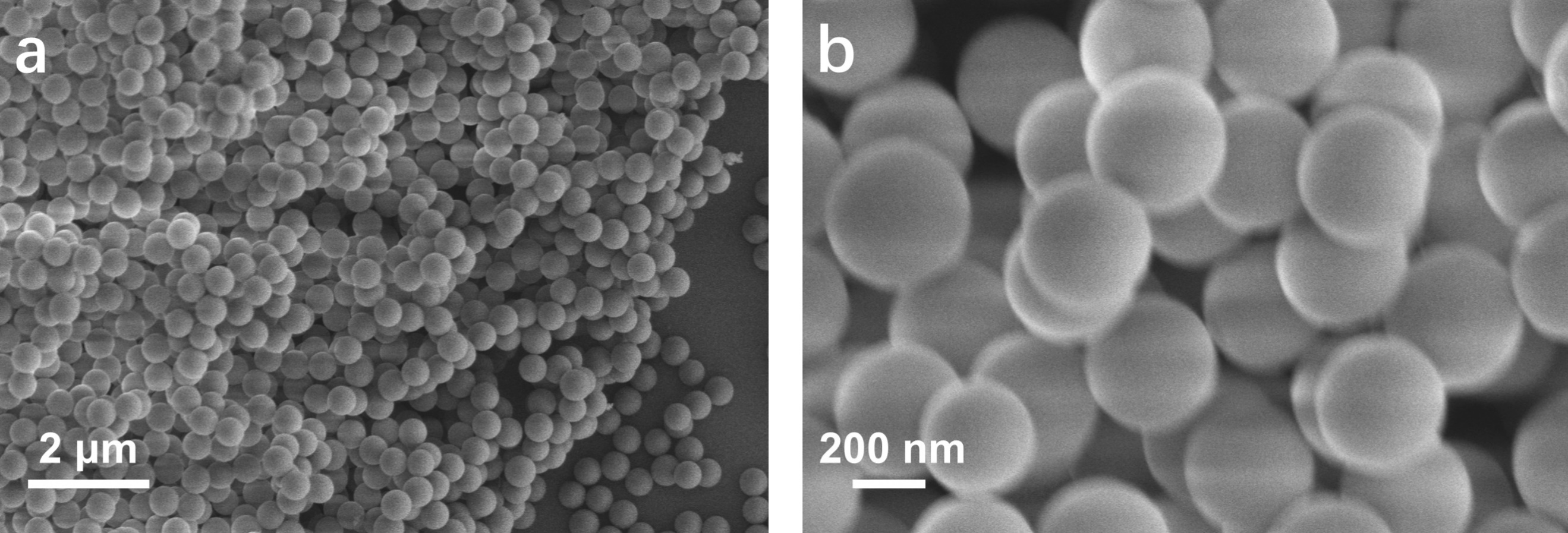


**Fig. S2** (a,b) FESEM images of SiO_2_ nanospheres.


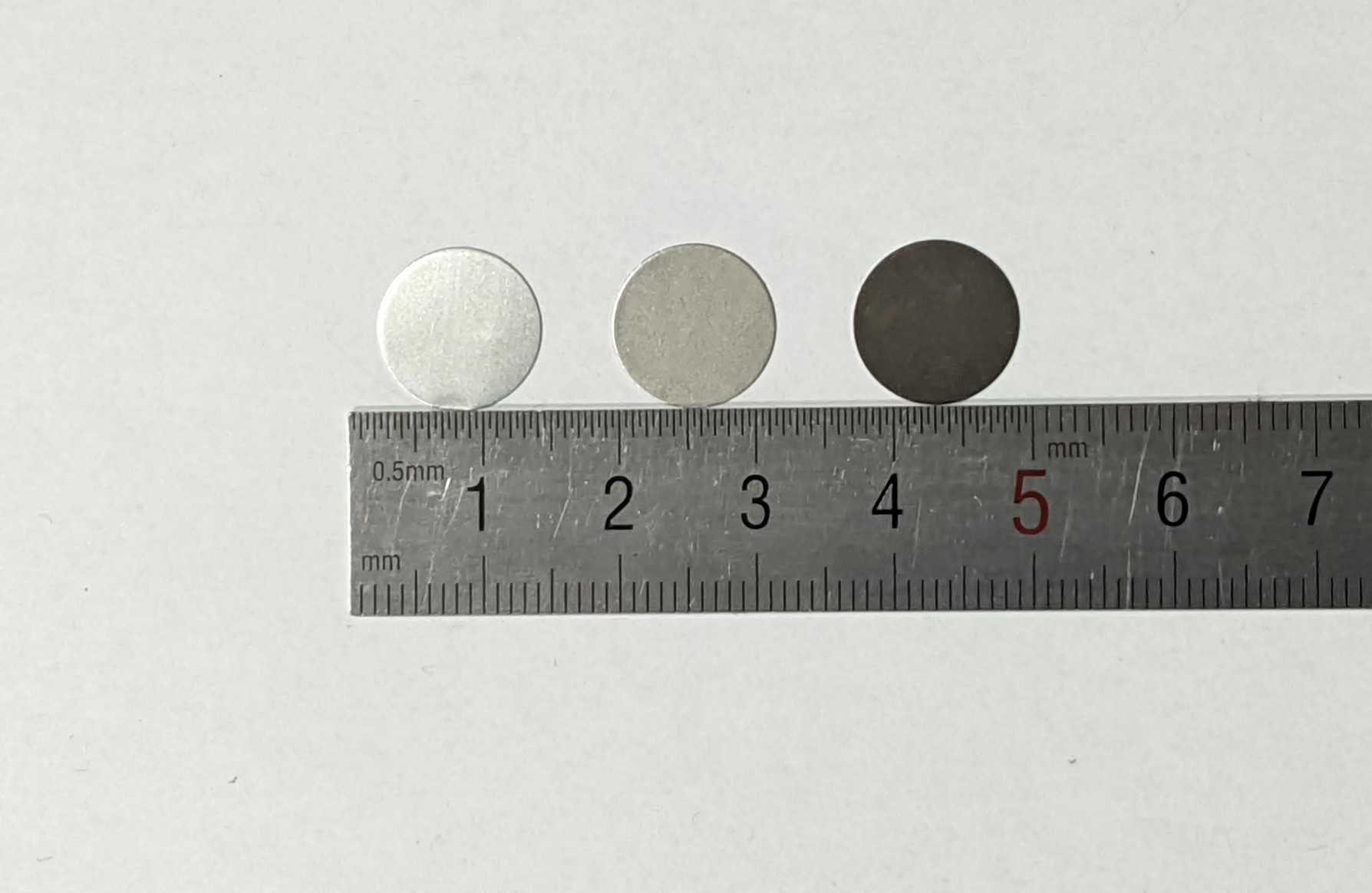


**Fig. S3** Optical images of pure Zn, Zn@ZnO and Zn@ZSCP.


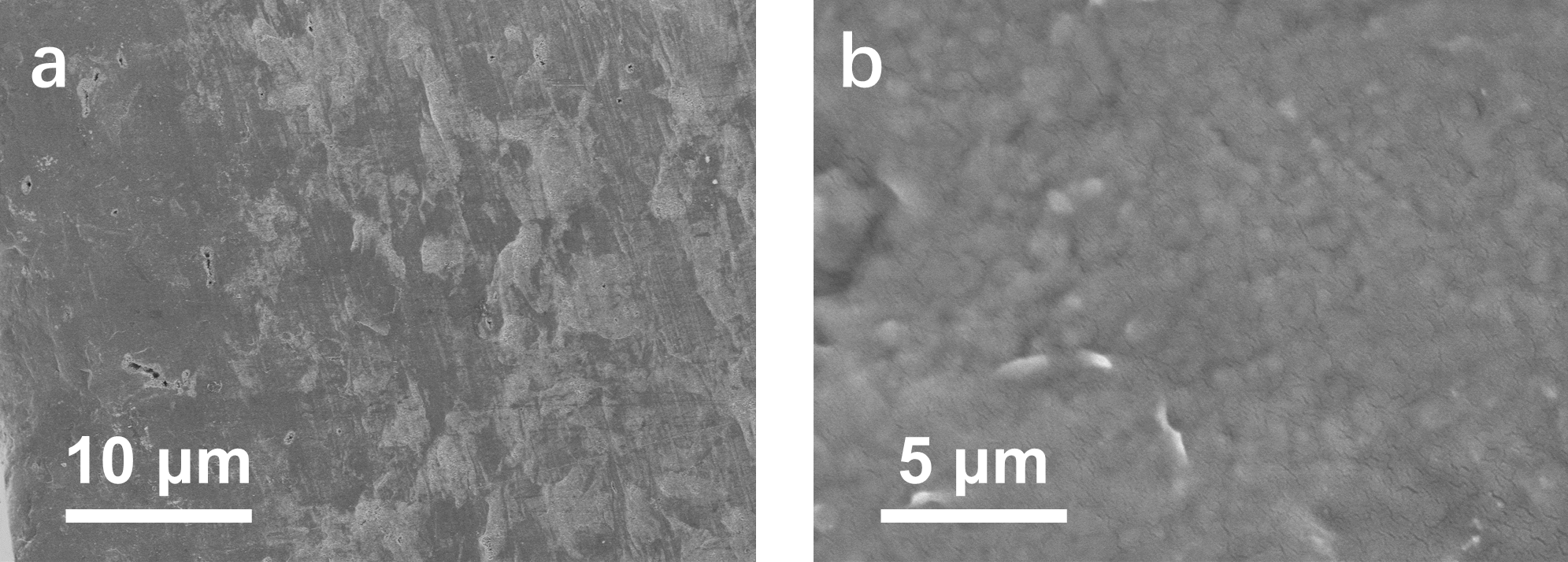


**Fig. S4** (a,b) FESEM images of Zn@ZnO.


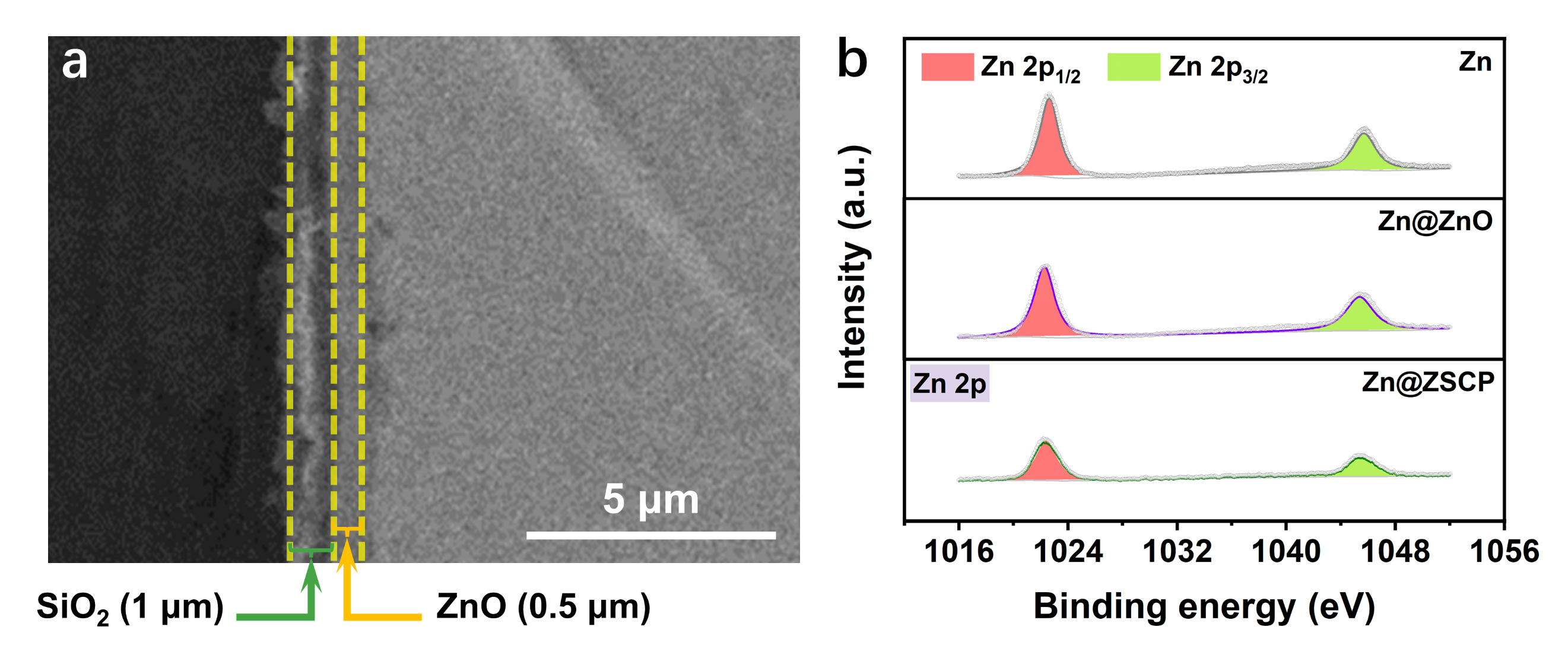


**Fig. S5** (a) The distribution and thickness of SiO_2_ and ZnO in ZSCP laye. (b) High resolution spectral Zn 2p XPS spectra.


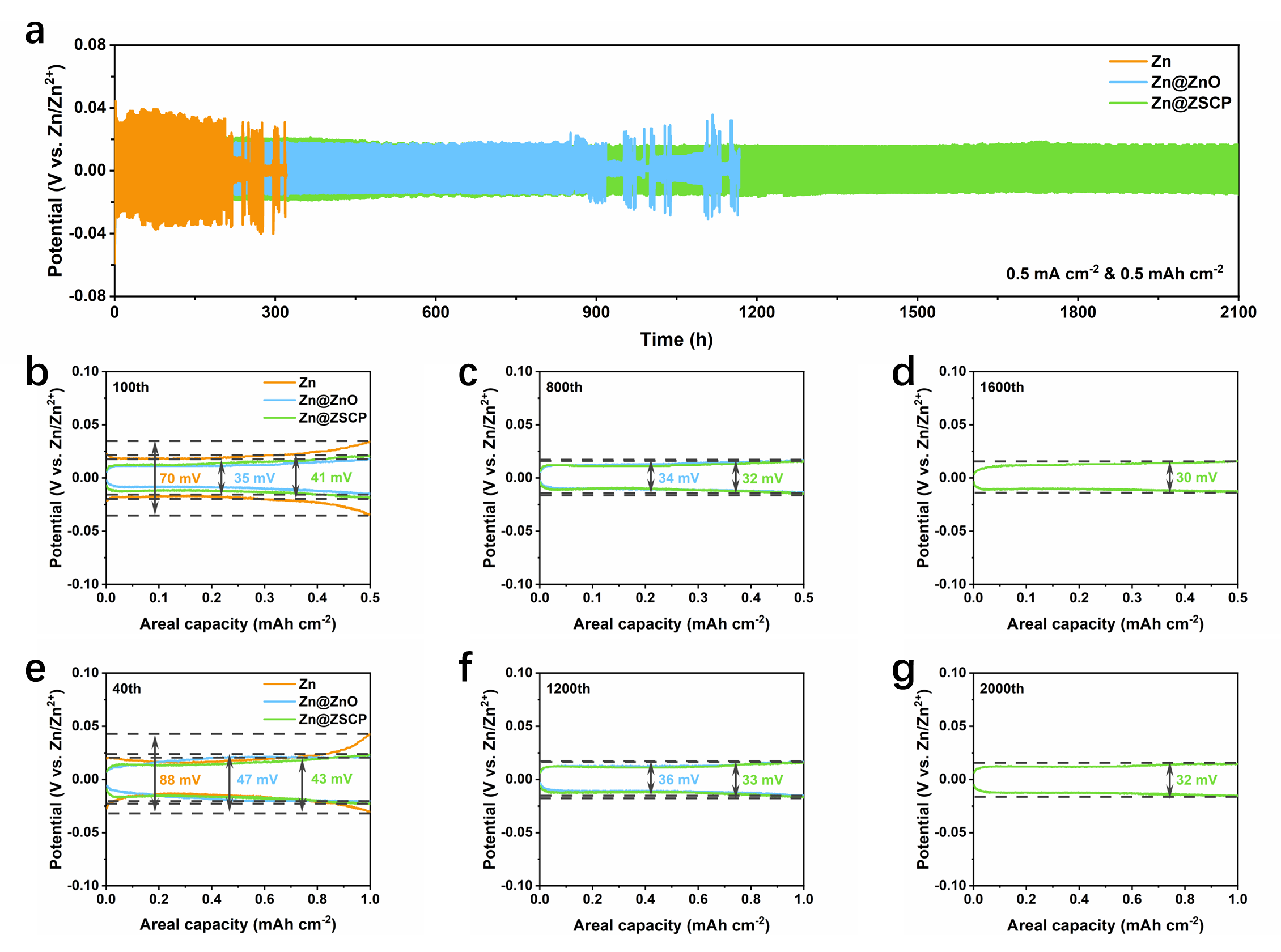


**Fig. S6** Electrochemical performance of the Zn and Zn@ZSCP symmetric cells. (a) Galvanostatic voltage curves of the symmetrical battery at an area current density of 0.5 mA cm^-2^ and an area capacitance of 0.5 mAh cm^-2^, as well as the corresponding constant current voltage curves at (b) 100th, (c) 800th, and (d) 1600th hour. The corresponding galvanostatic voltage curves at (e) 40th, (f) 1200th, and (g) 2000th hour at an area current density of 1 mA cm^-2^ and an area capacitance of 1 mAh cm^-2^.


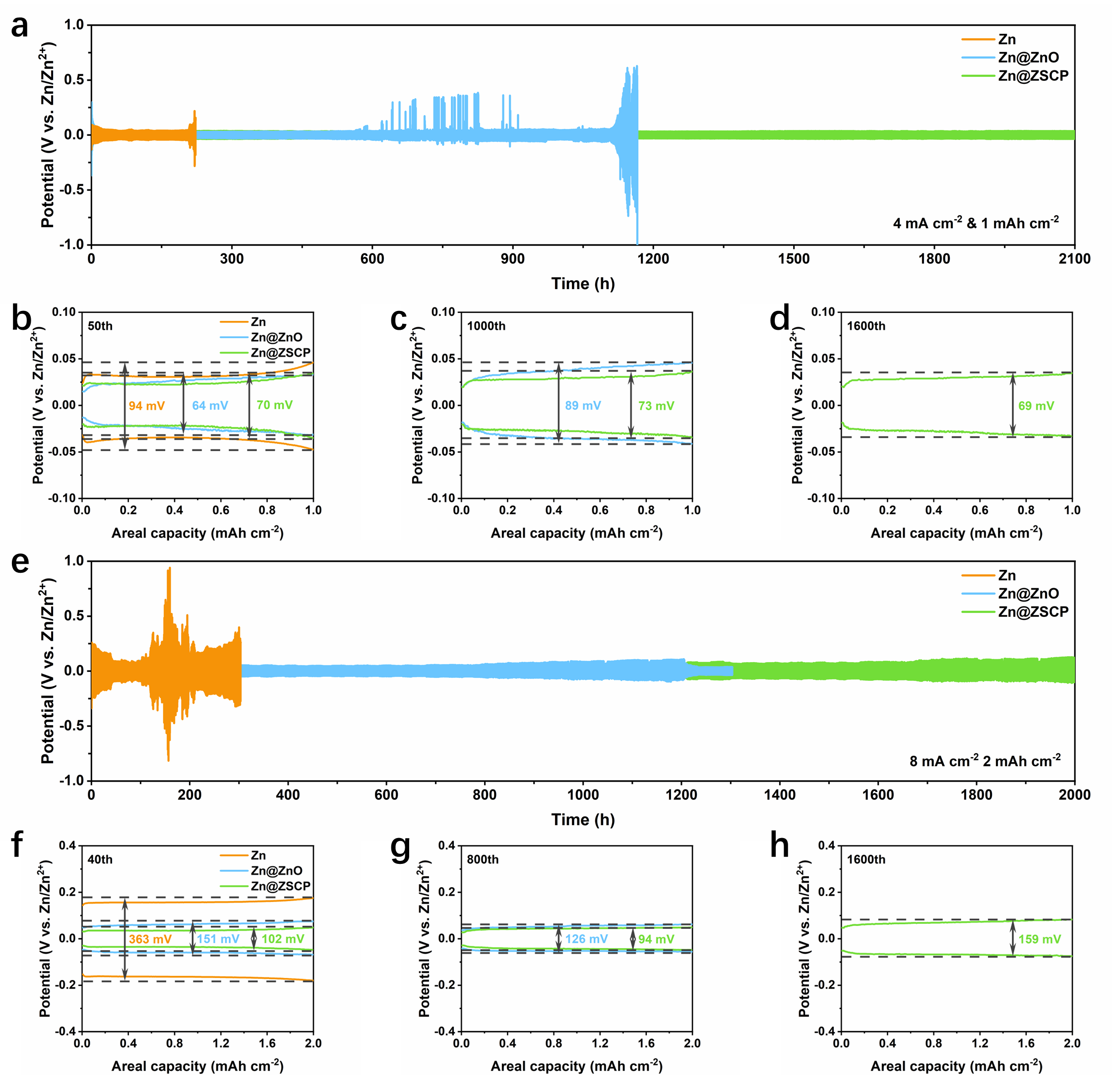


**Fig. S7** Electrochemical performance of the Zn and Zn@ZSCP symmetric cells. (a) Galvanostatic voltage curves of the symmetrical battery at an area current density of 4 mA cm^-2^ and an area capacitance of 1 mAh cm^-2^, as well as the corresponding constant current voltage curves at (b) 50th, (c) 1000th, and (d) 1600th hour. (e) Galvanostatic voltage curves of the symmetrical battery at an area current density of 8 mA cm^-2^ and an area capacitance of 2 mAh cm^-2^, as well as the corresponding galvanostatic voltage curves at (f) 40th, (g) 800th, and (h) 1600th hour.





**Fig. S8** Cycle nucleation potential of Zn, Zn@ZnO and Zn@ZSCP symmetric cells at different current densities.


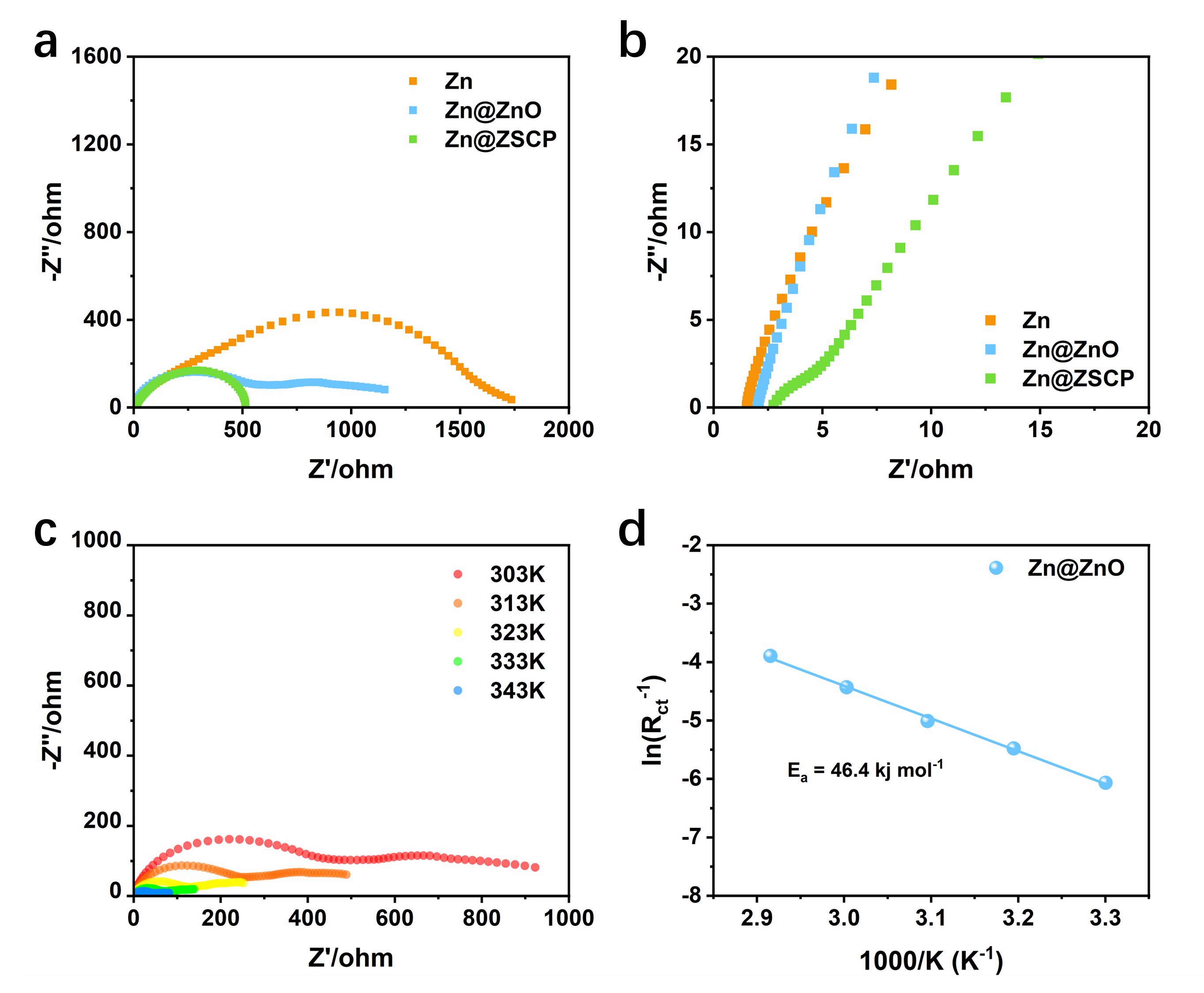


**Fig. S9** (a) Nyquist plots and (b) that in high frequency region Nyquist plots of symmetrical cells before cycling. The cells were tested after a night of standing. (c) Nyquist electrochemical impedance spectroscopy (EIS) plots at different temperatures of 30-70℃ before cycling. (d) Arrhenius curves and comparison of activation energies (Ea) of Zn@ZnO electrodes.


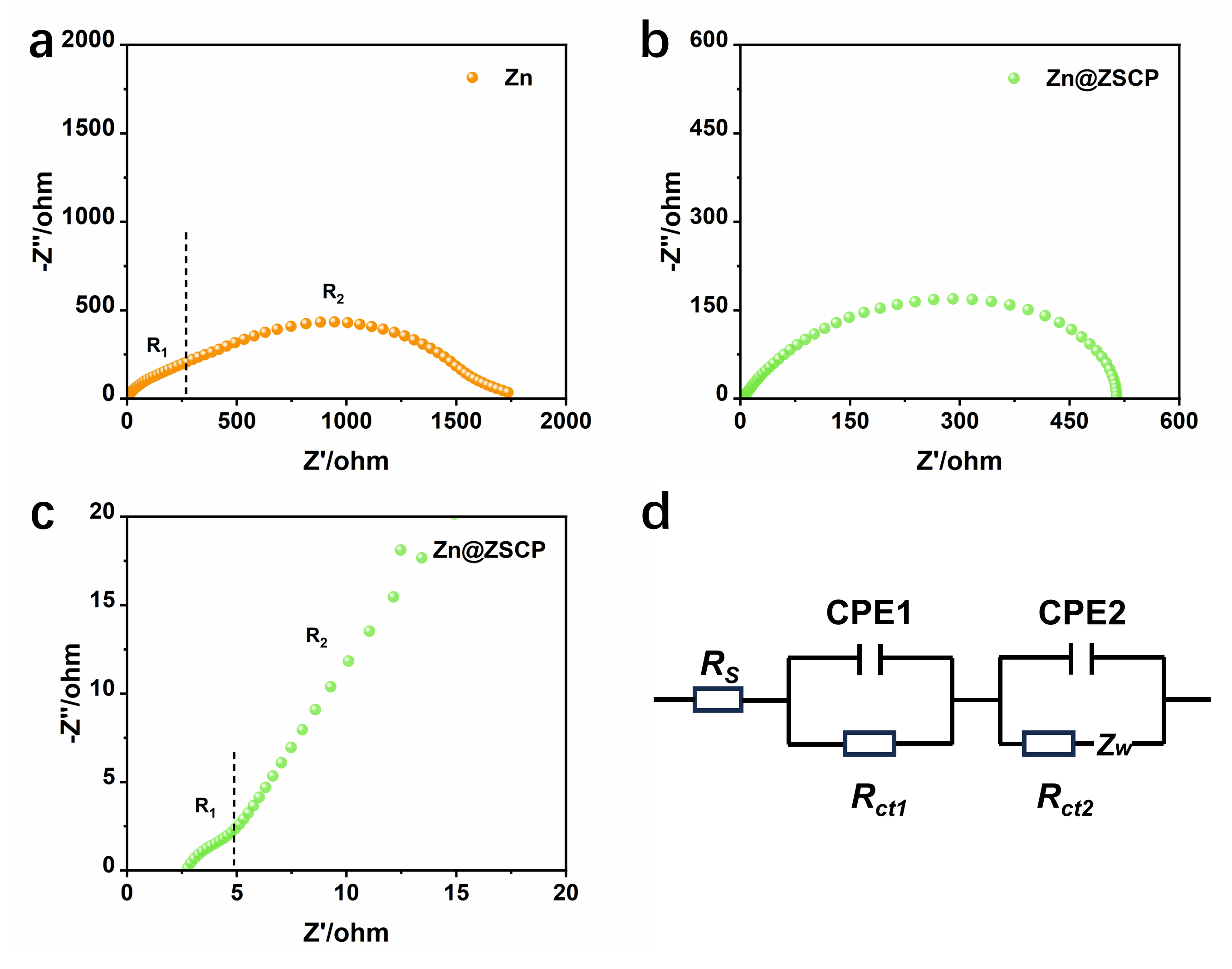


**Fig. S10** (a) The Nyquist plot of Zn and Zn@ZSCP symmetric cells and (b) the corresponding equivalent circuit models. The *R_s_* represents the resistance of electrolyte, and *R_ct1_* at the high frequency and *R_ct2_* at the low frequency correspond to the charge transfer resistance.


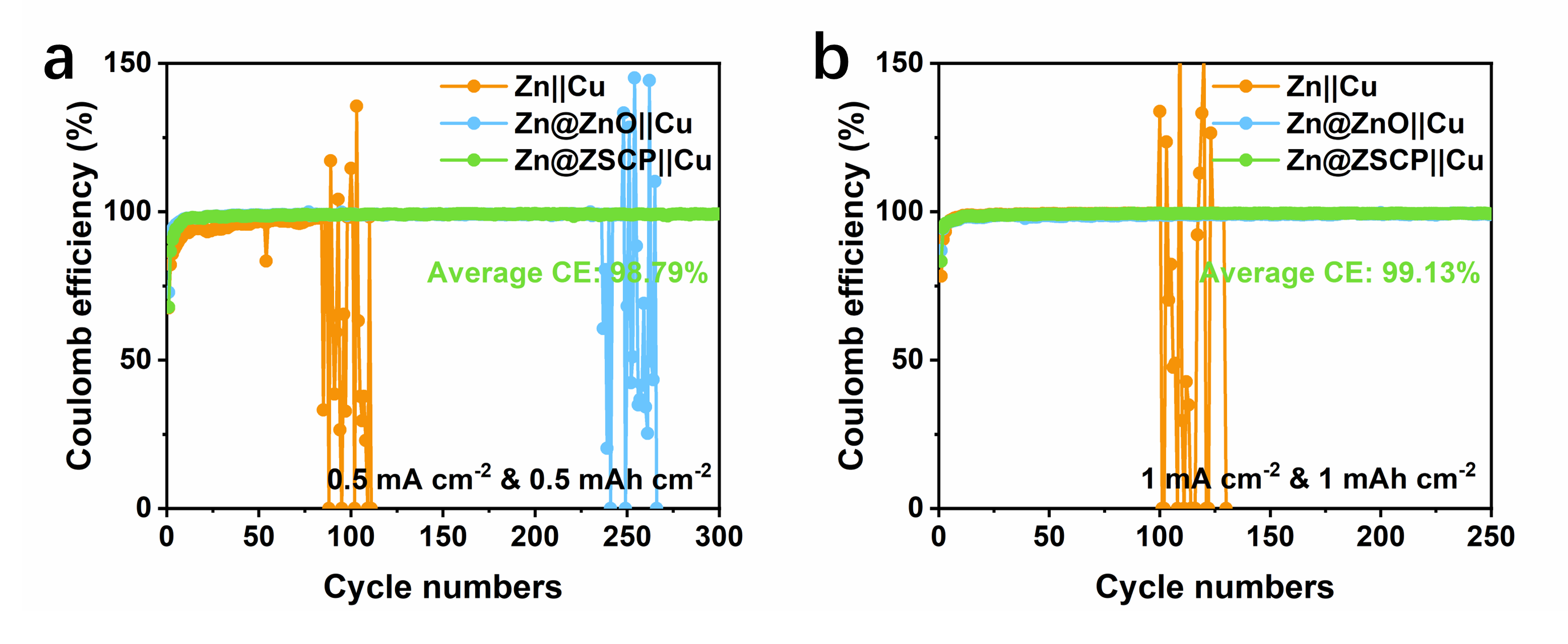


**Fig. S11** Coulombic efficiency of pure Zn//Cu, Zn@ZnO//Cu and Zn@ZSCP//Cu asymmetrical cells, (a) 0.5 mA cm^-2^ and 0.5 mAh cm^-2^, (b) 1 mA cm^-2^ and 1 mAh cm^-2^.


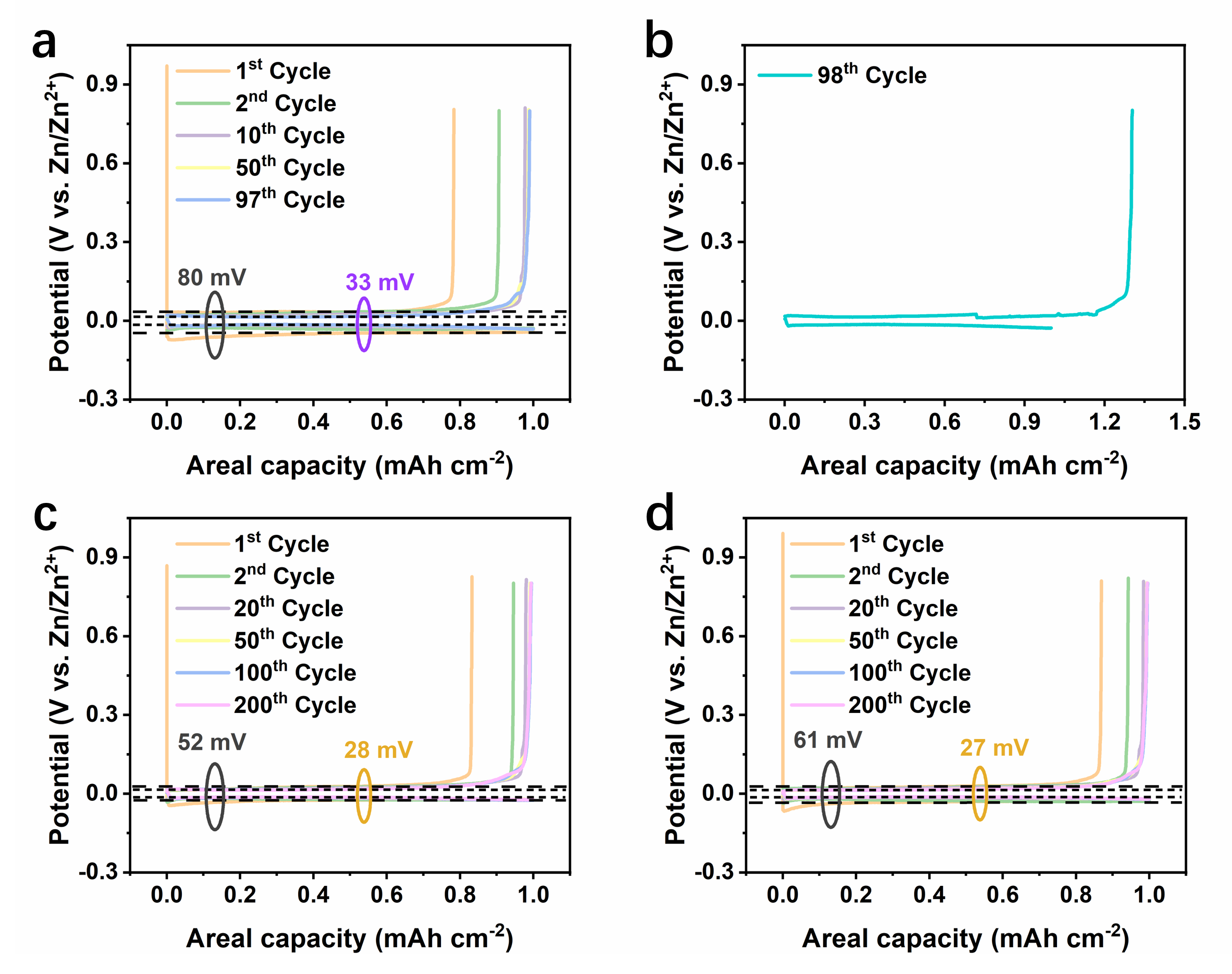


**Fig. S12** Galvanostatic voltage curves at 1 mA cm^-2^ and 1 mAh cm^-2^ of (a-b) pure Zn//Cu, (c) Zn@ZnO//Cu and (d) Zn@ZSCP//Cu asymmetrical cells.


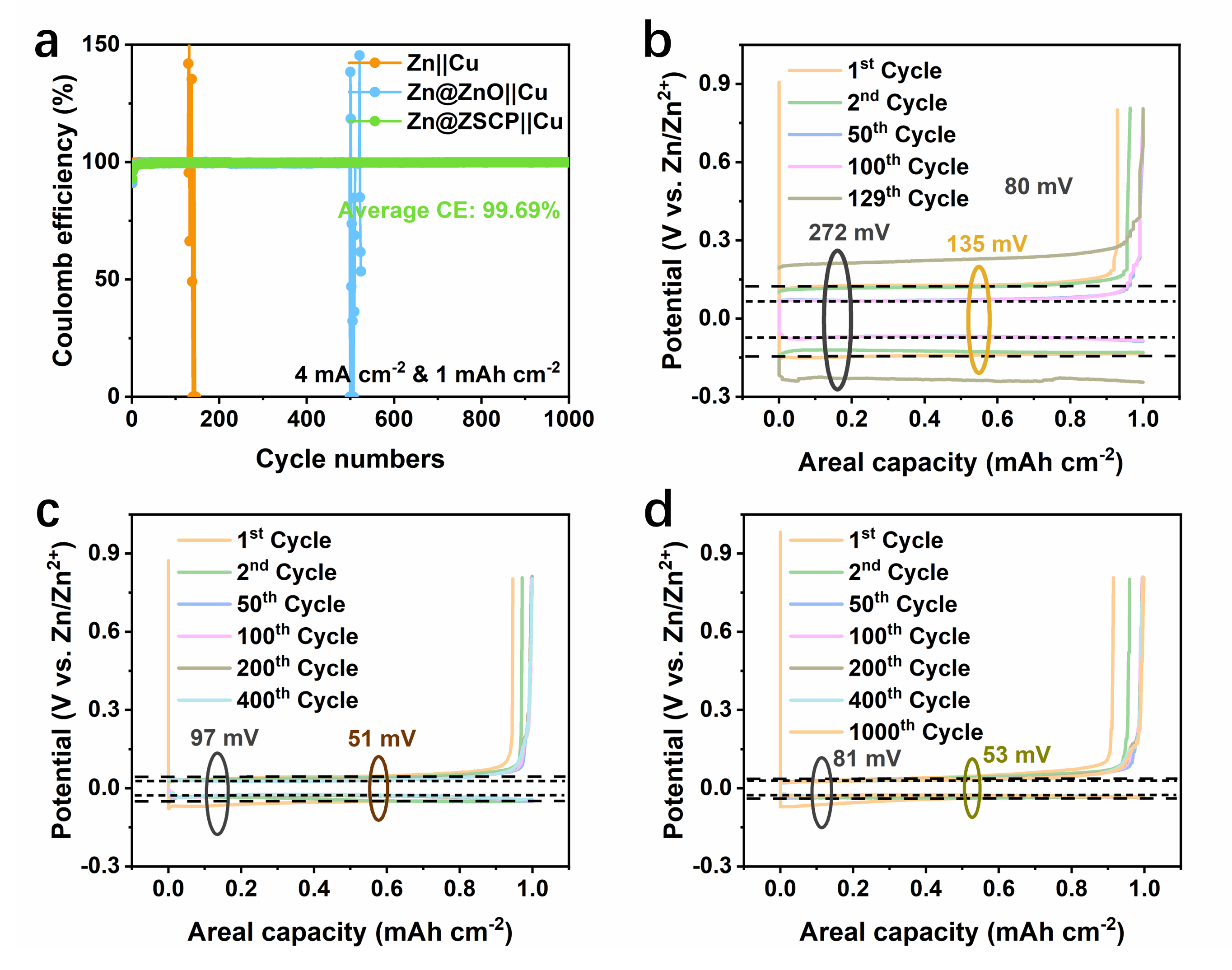


**Fig. S13** (a) Coulombic efficiency and corresponding galvanostatic voltage curves at 4 mA cm^-2^ and 1 mAh cm^-2^ of (b) pure Zn//Cu, (c) Zn@ZnO//Cu and (d) Zn@ZSCP//Cu asymmetrical cells.





**Fig. S14** Nucleation potential of at various current densities of pure Zn//Cu, Zn@ZnO//Cu and Zn@ZSCP//Cu asymmetrical cells.





**Fig. S15** CV curves of Zn plating/stripping of asymmetrical cells at 0.1 mV s^-1^.


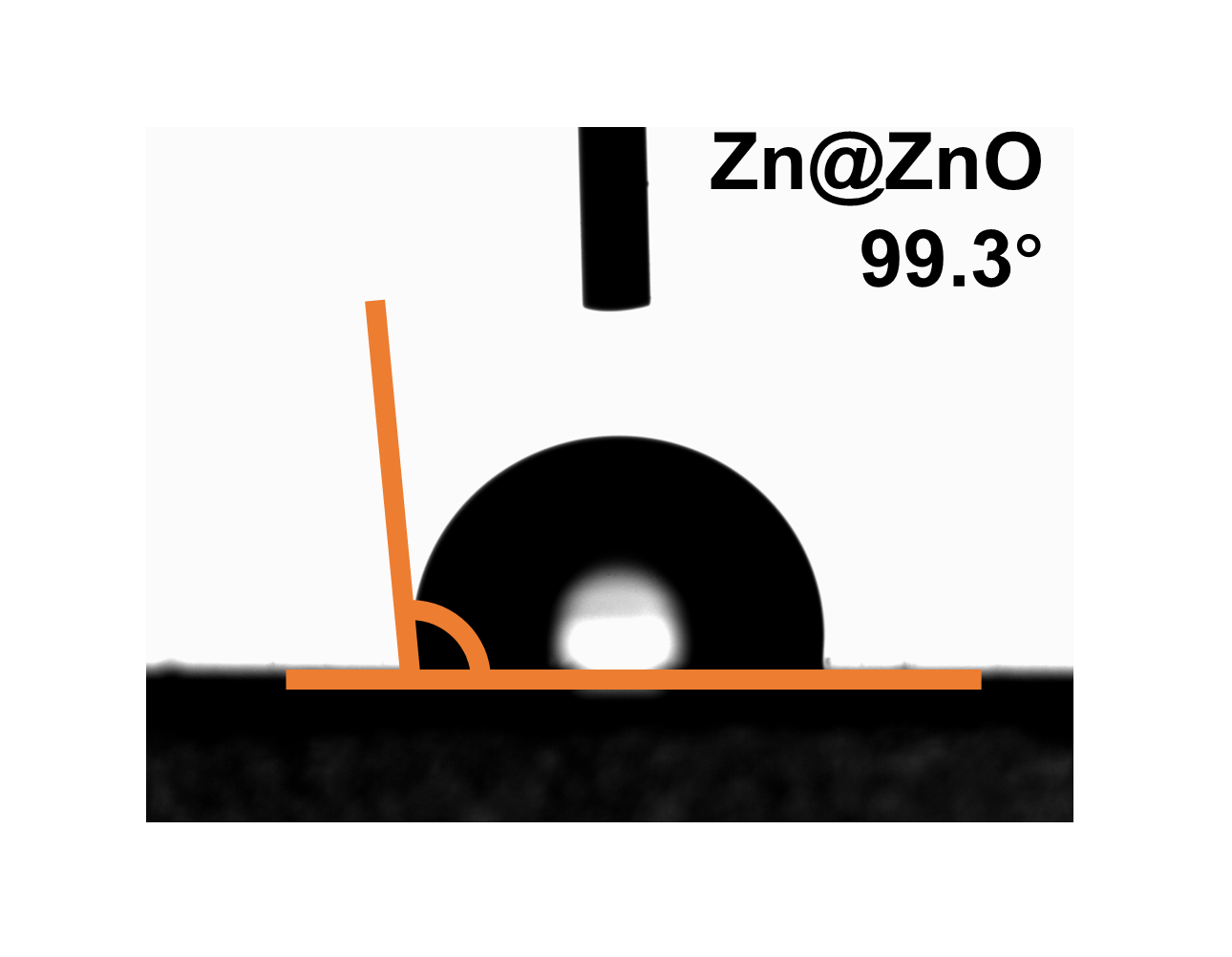


**Fig. S16** Contact angle test of Zn@ZnO.


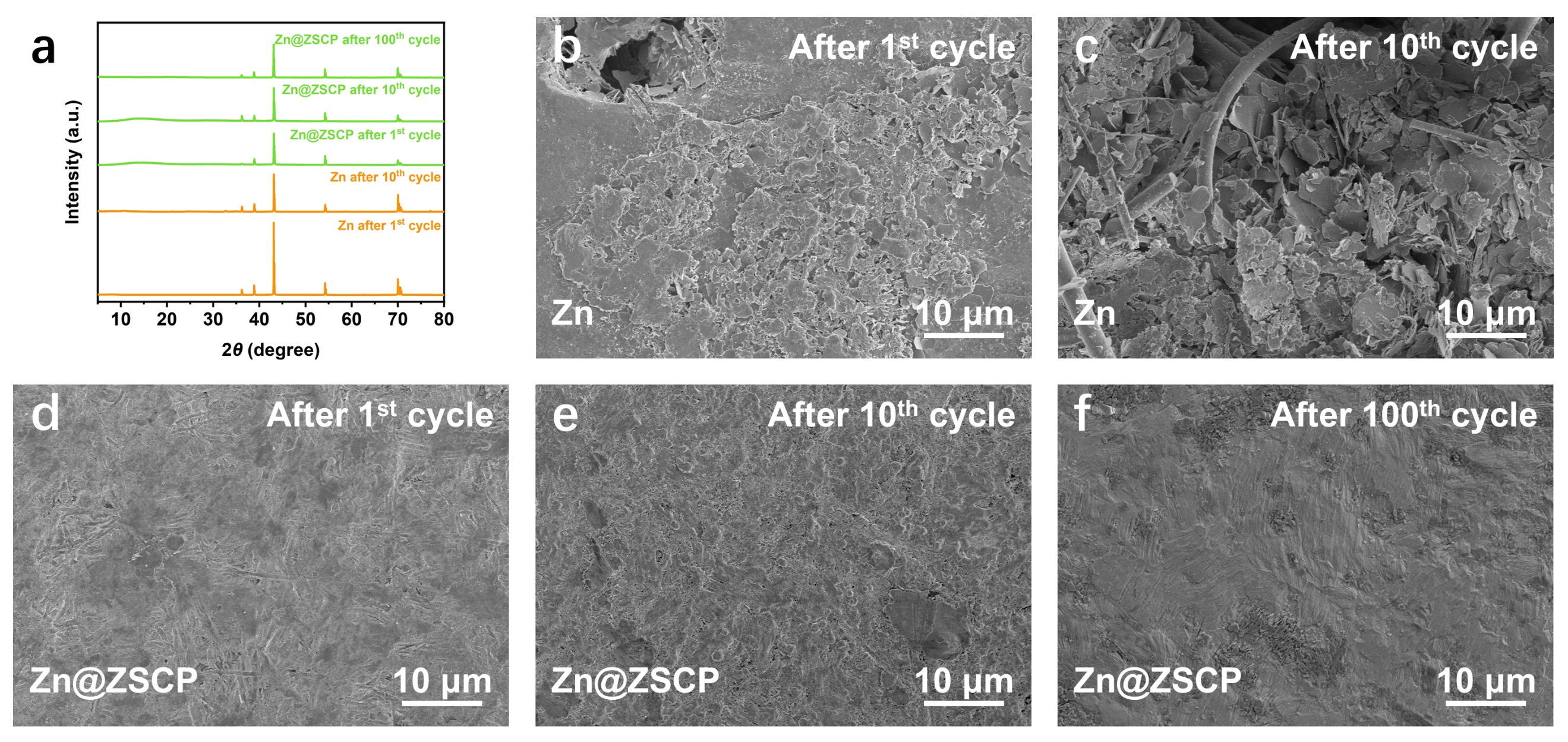


**Fig. S17** (a) The XRD patterns of pure Zn and Zn@ZSCP after deposition at 1 mA cm^-2^ and 1 mAh cm^-2^ for 1 and 10 cycles. FESEM images of (b-c) pure Zn and (d-f) Zn@ZSCP after deposition at 1 mA cm^-2^ and 1 mAh cm^-2^ for 1 and 10 cycles.


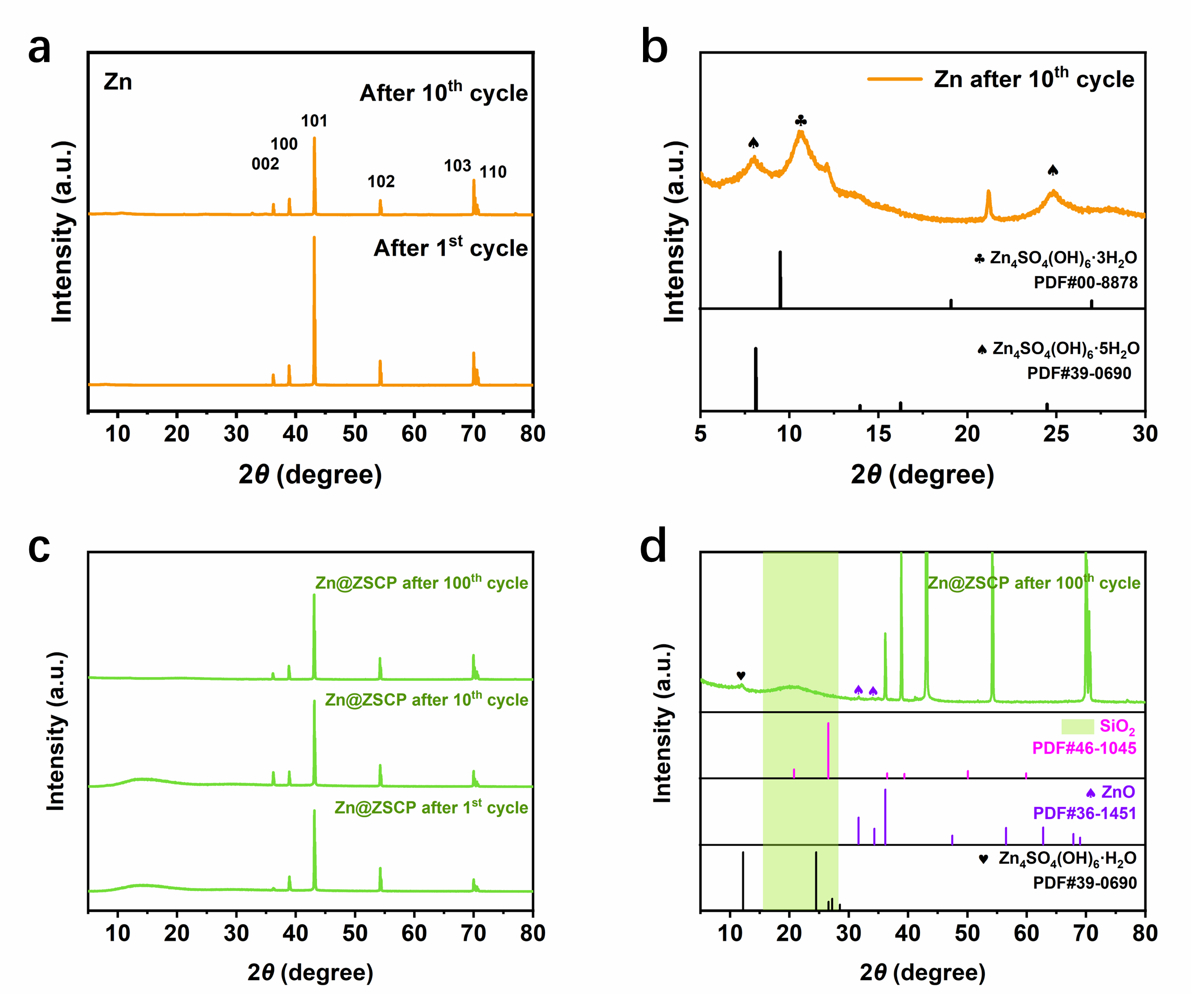


**Fig. S18** XRD patterns of (a-b) pure Zn and (c-d) Zn@ZSCP after 10 cycles.


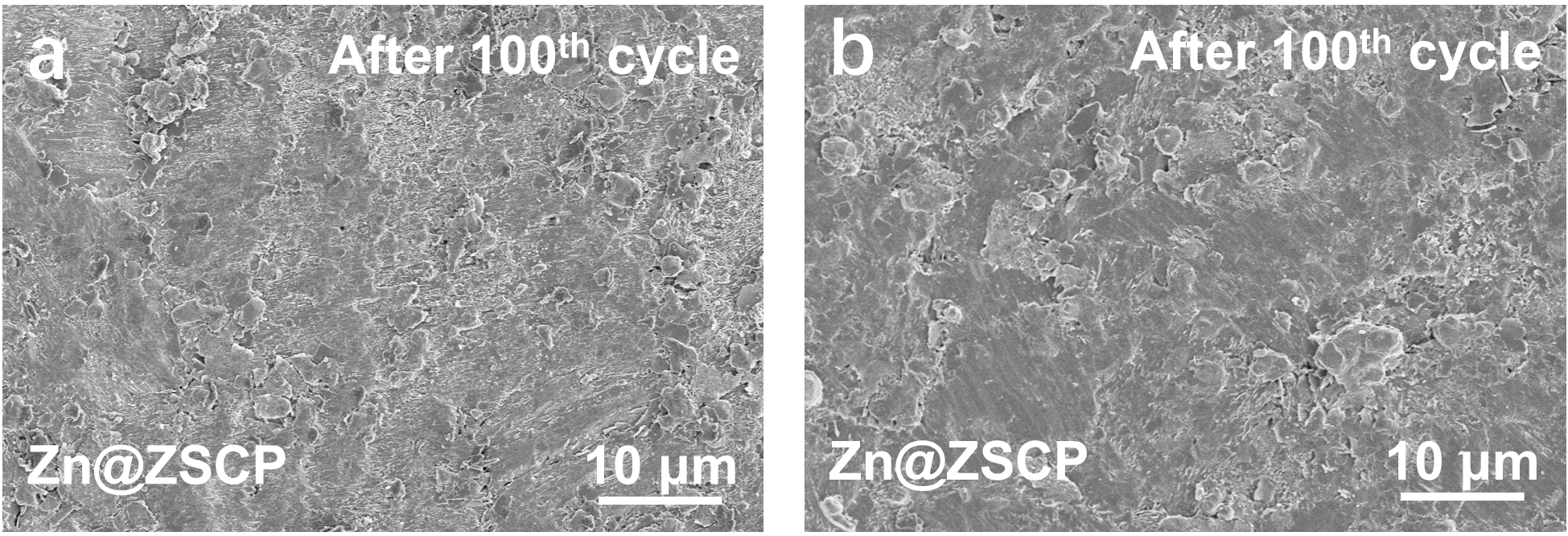


**Fig. S19** FESEM images of Zn@ZnO after deposition at 1 mA cm^-2^ and 1 mAh cm^-2^ for 1 and 10 cycles.


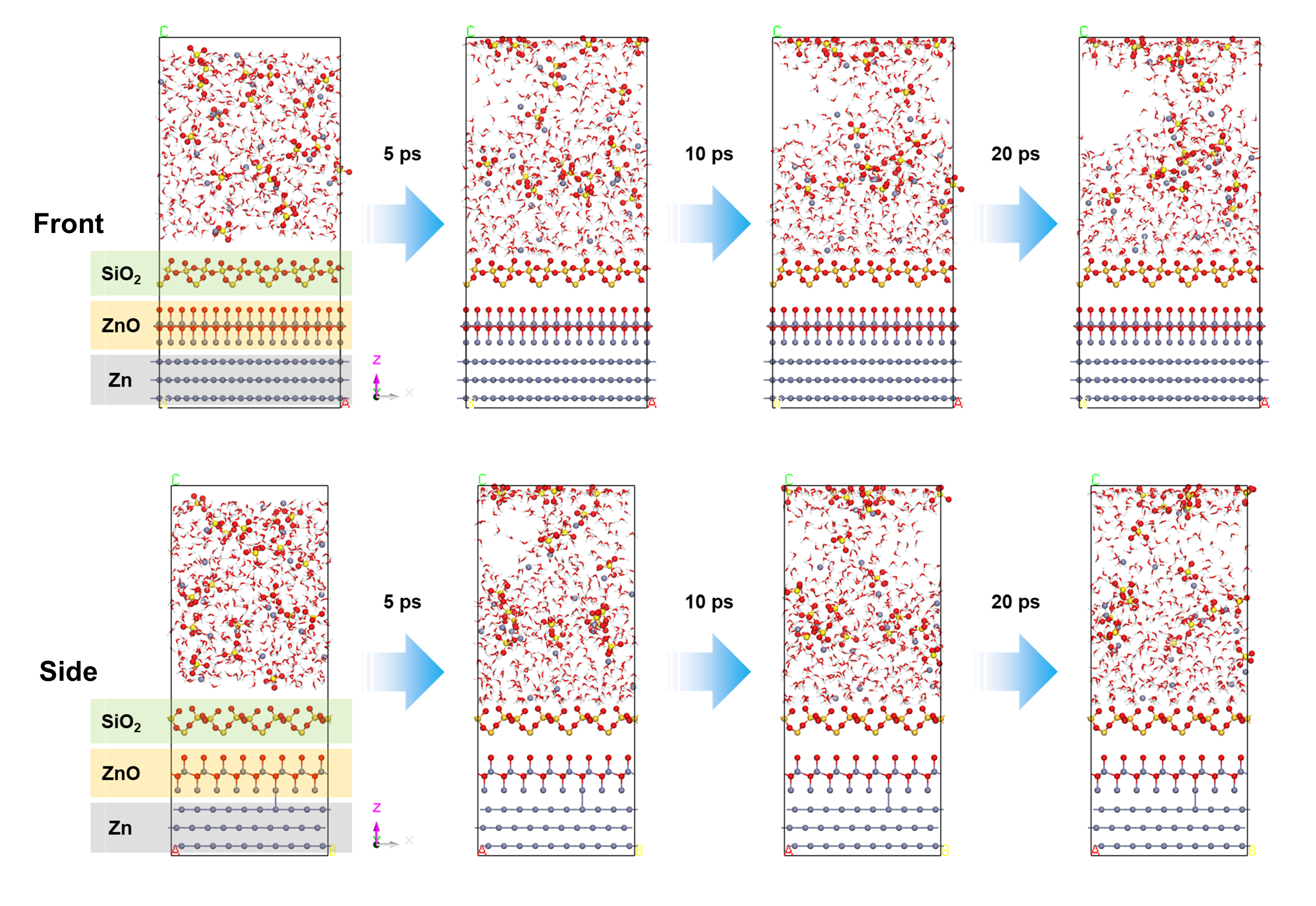


**Fig. S20** MD simulation snapshot (side view) of Zn^2+^ ions and H_2_O molecule on the surface of Zn@ZSCP composite foil in 2M ZnSO_4_ aqueous electrolyte.


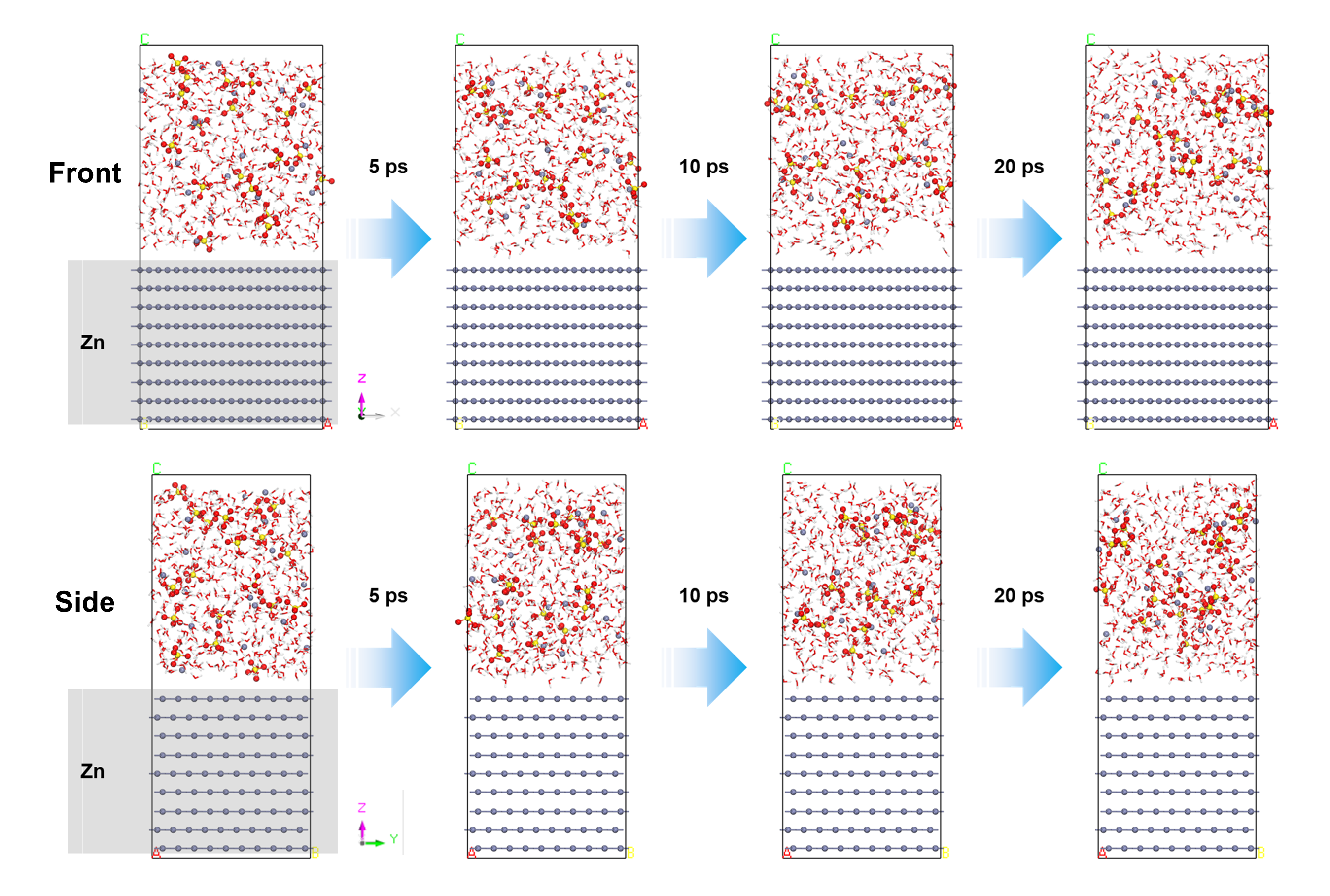


**Fig. S21** MD simulation snapshot of Zn^2+^ ions and H_2_O molecule on the surface of Zn foil in 2M ZnSO_4_ aqueous electrolyte. (a) front view, (a) side view.


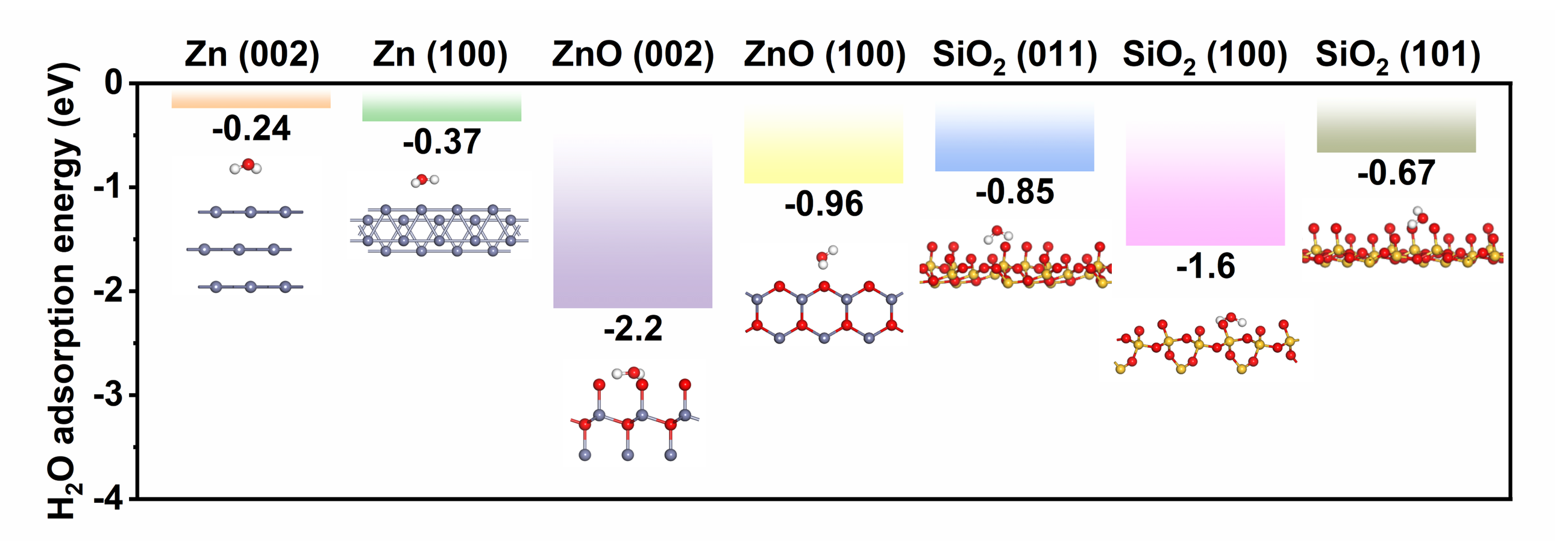


**Fig. S22** The adsorption of H_2_O molecules on Zn and SiO_2_.


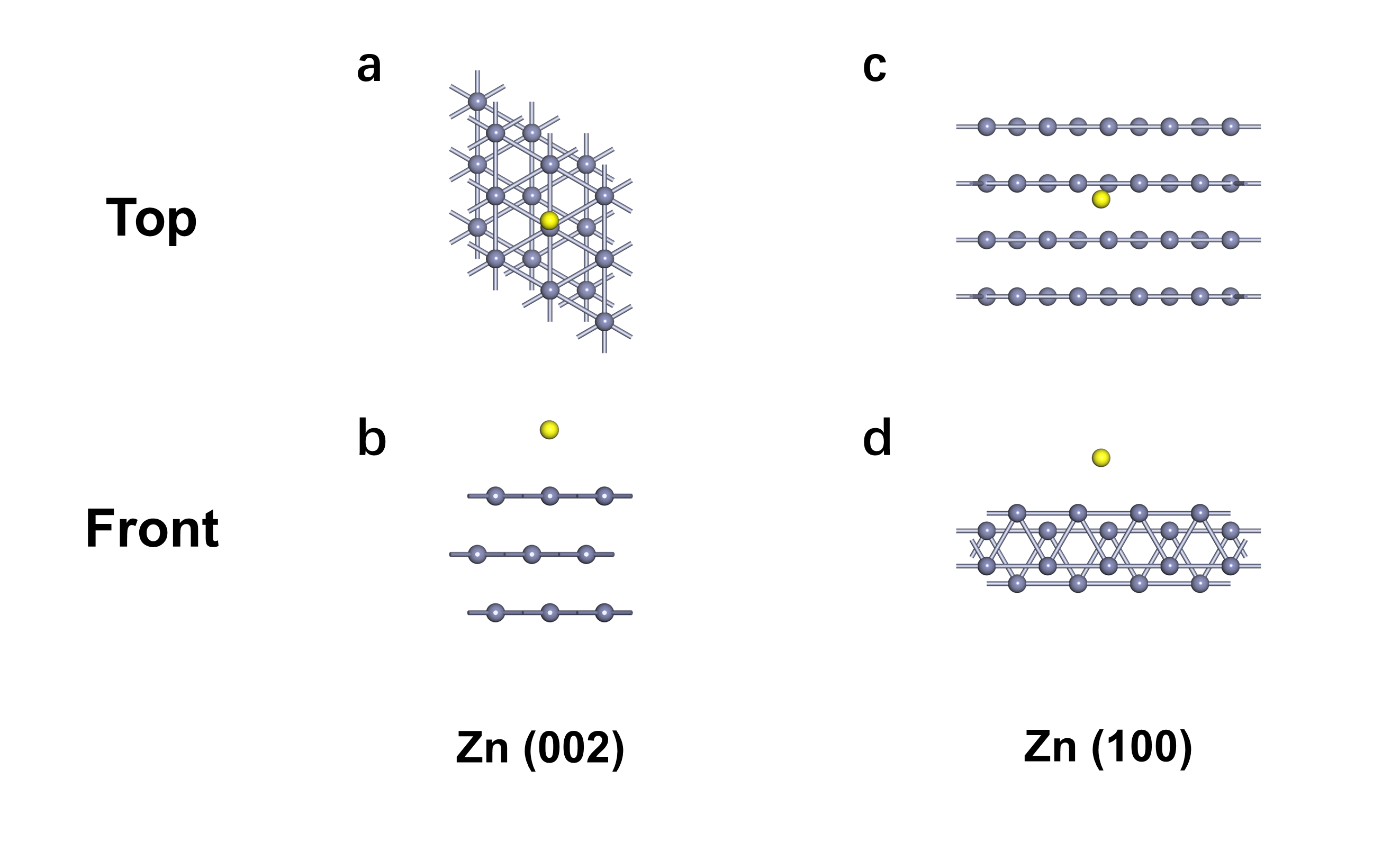


**Fig. S23** The adsorption sites of Zn^2+^ in Zn (002) and Zn (100) lattices.


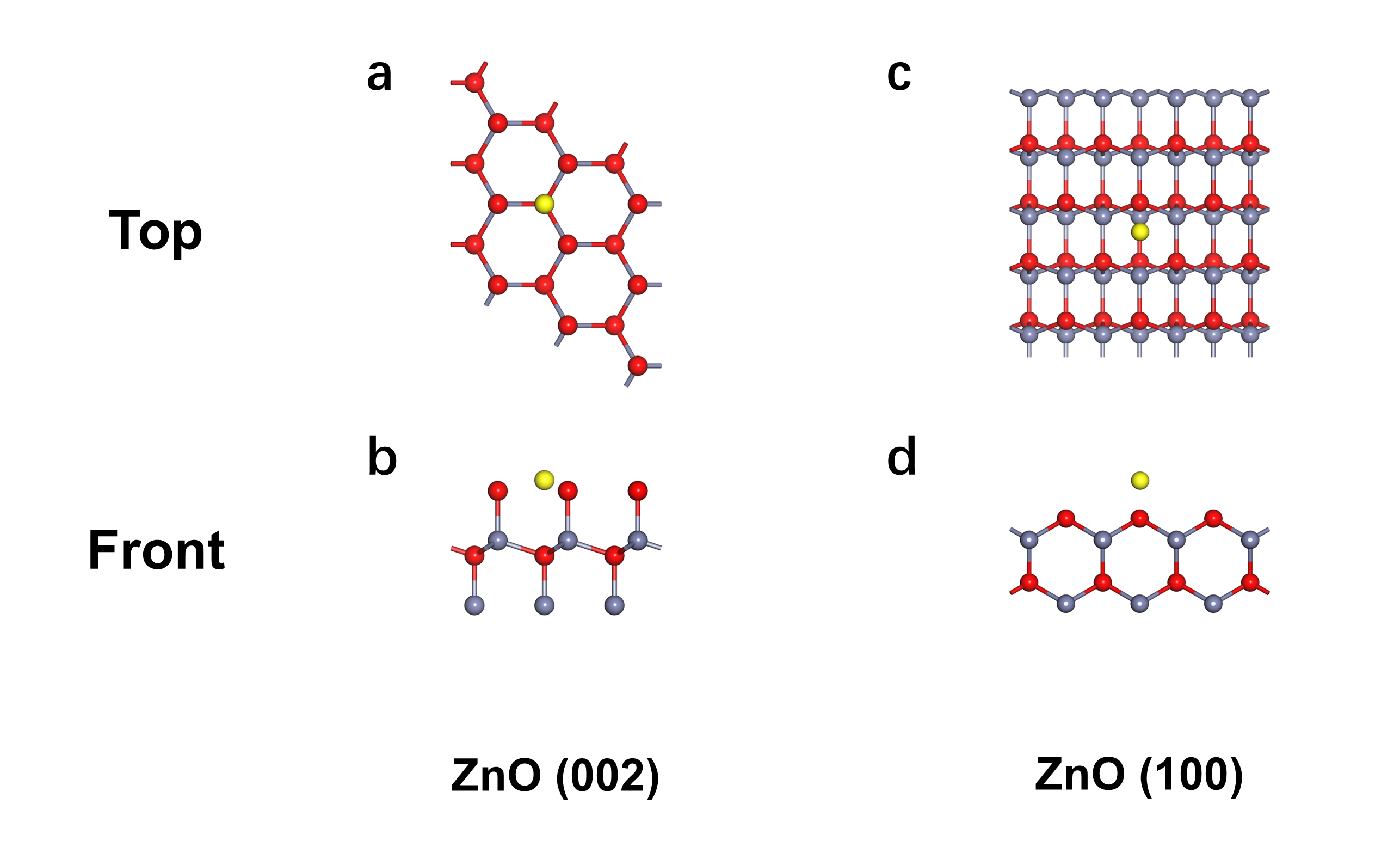


**Fig. S24** The adsorption sites of Zn^2+^ in ZnO (002) and ZnO (100) lattices.


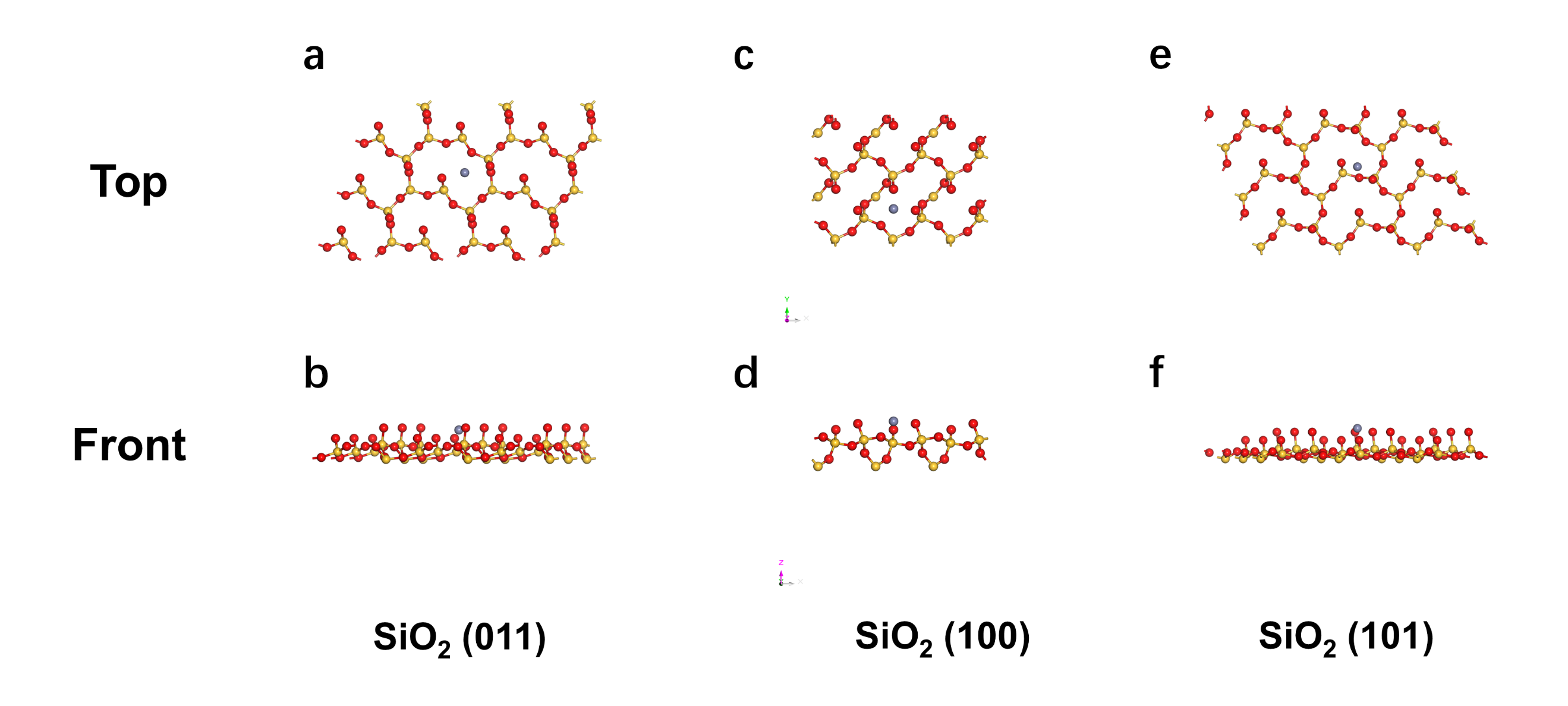


**Fig. S25** The adsorption sites of Zn^2+^ in SiO_2_ (011), SiO_2_ (100) and SiO_2_ (101) lattices.


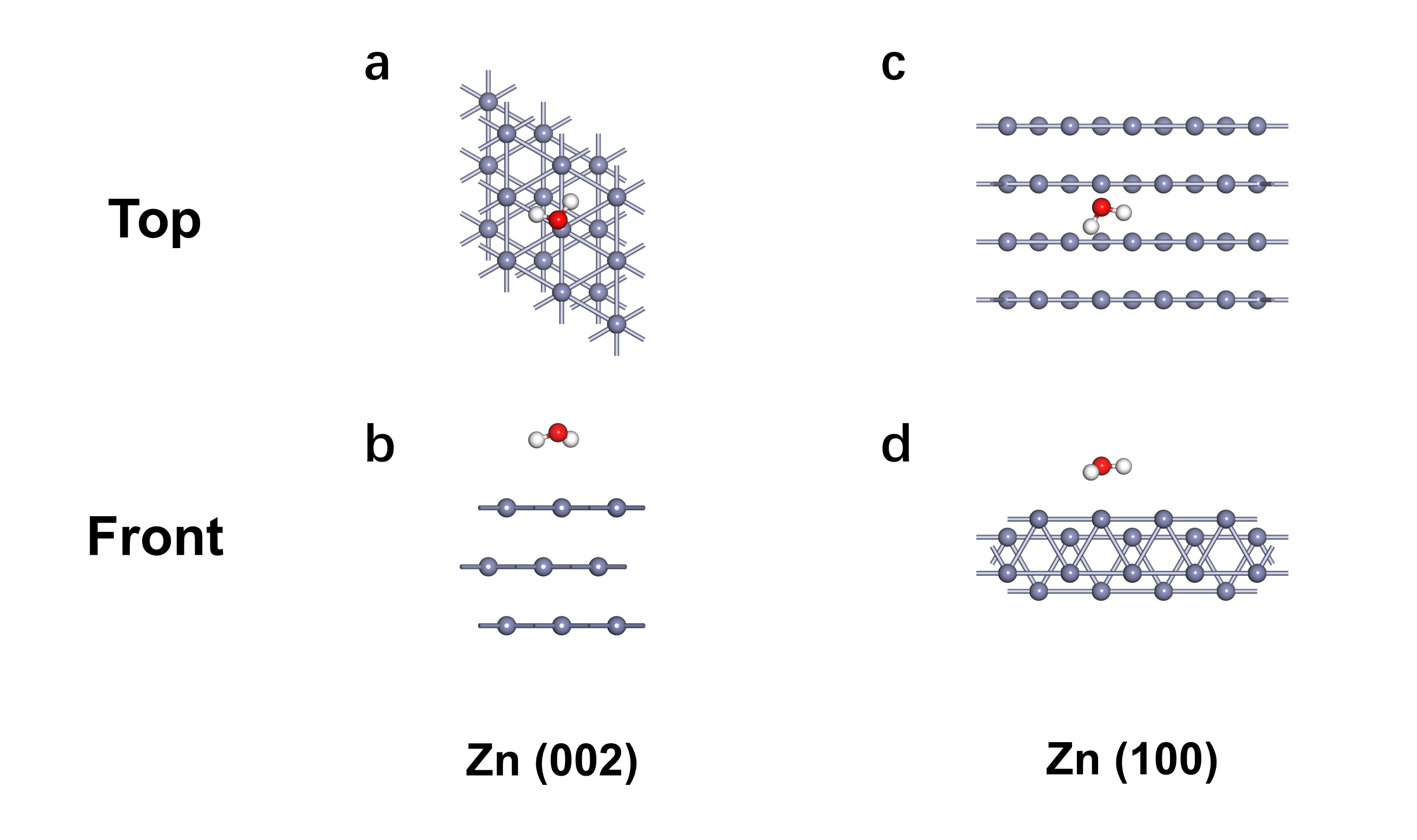


**Fig. S26** The adsorption sites of H_2_O molecule in Zn (002) and Zn (100) lattices.


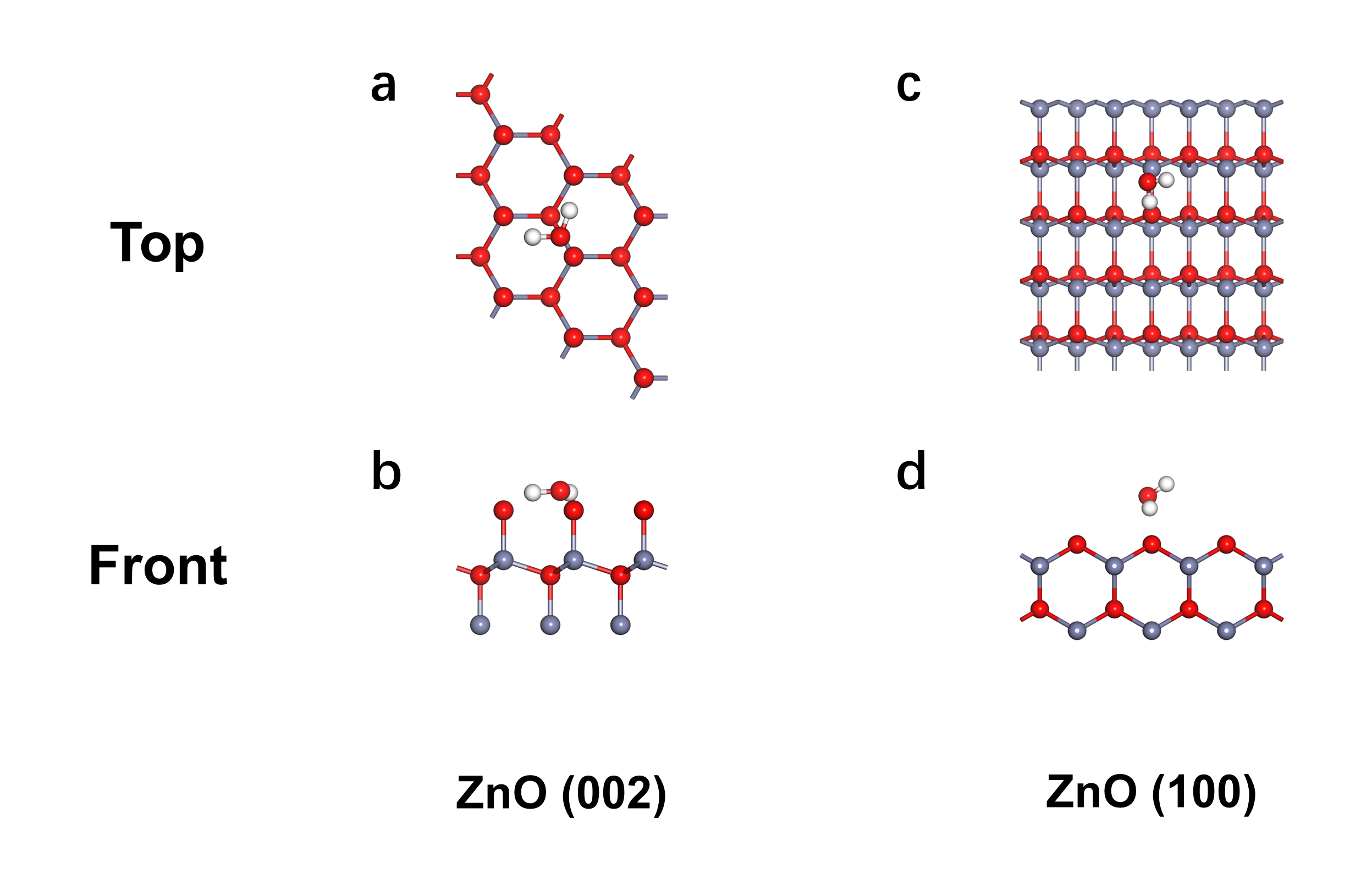


**Fig. S27** The adsorption sites of H_2_O molecule in ZnO (002) and ZnO (100) lattices.


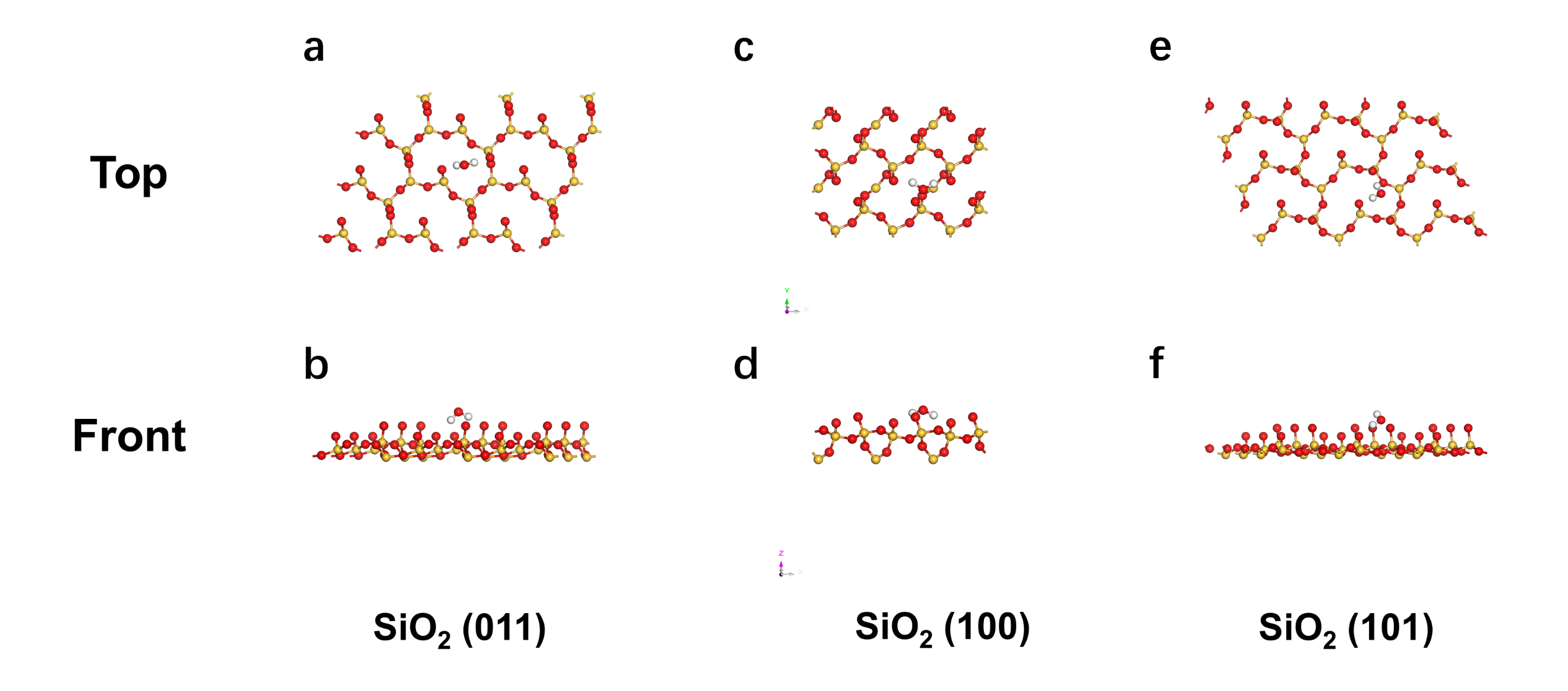


**Fig. S28** The adsorption sites of H_2_O molecule in SiO_2_ (011), SiO_2_ (100) and SiO_2_ (101) lattices.





**Fig. S29** The hydrogen adsorption Gibbs free energy for HER in Zn (002) and Zn (100) lattices.





**Fig. S30** The hydrogen adsorption Gibbs free energy for HER in ZnO (002) and ZnO (100) lattices.





**Fig. S31** The hydrogen adsorption Gibbs free energy for HER in SiO_2_ (011), SiO_2_ (100), and SiO_2_ (101) lattices.


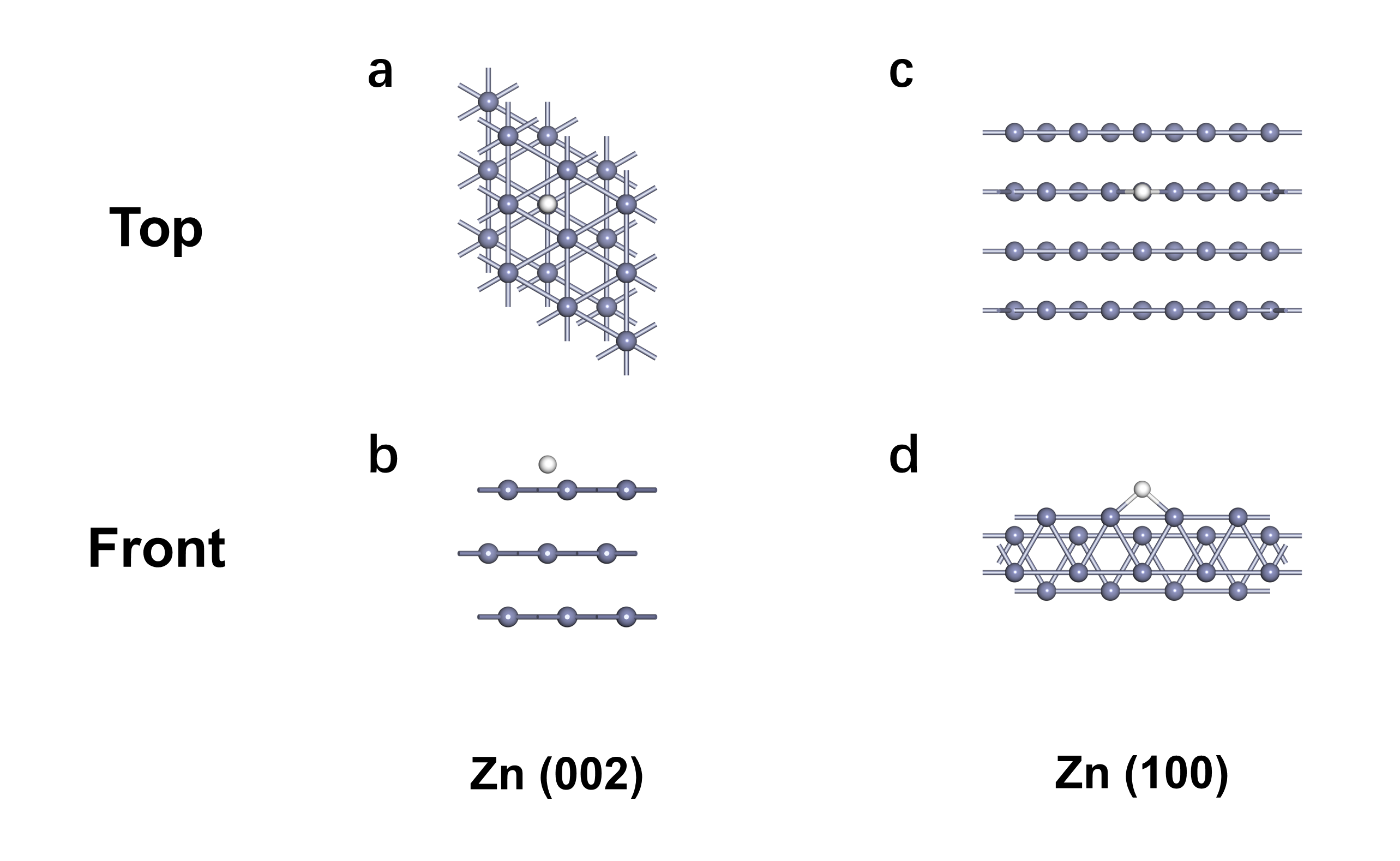


**Fig. S32** The adsorption sites of hydrogen proton in Zn (002) and Zn (100) lattices.


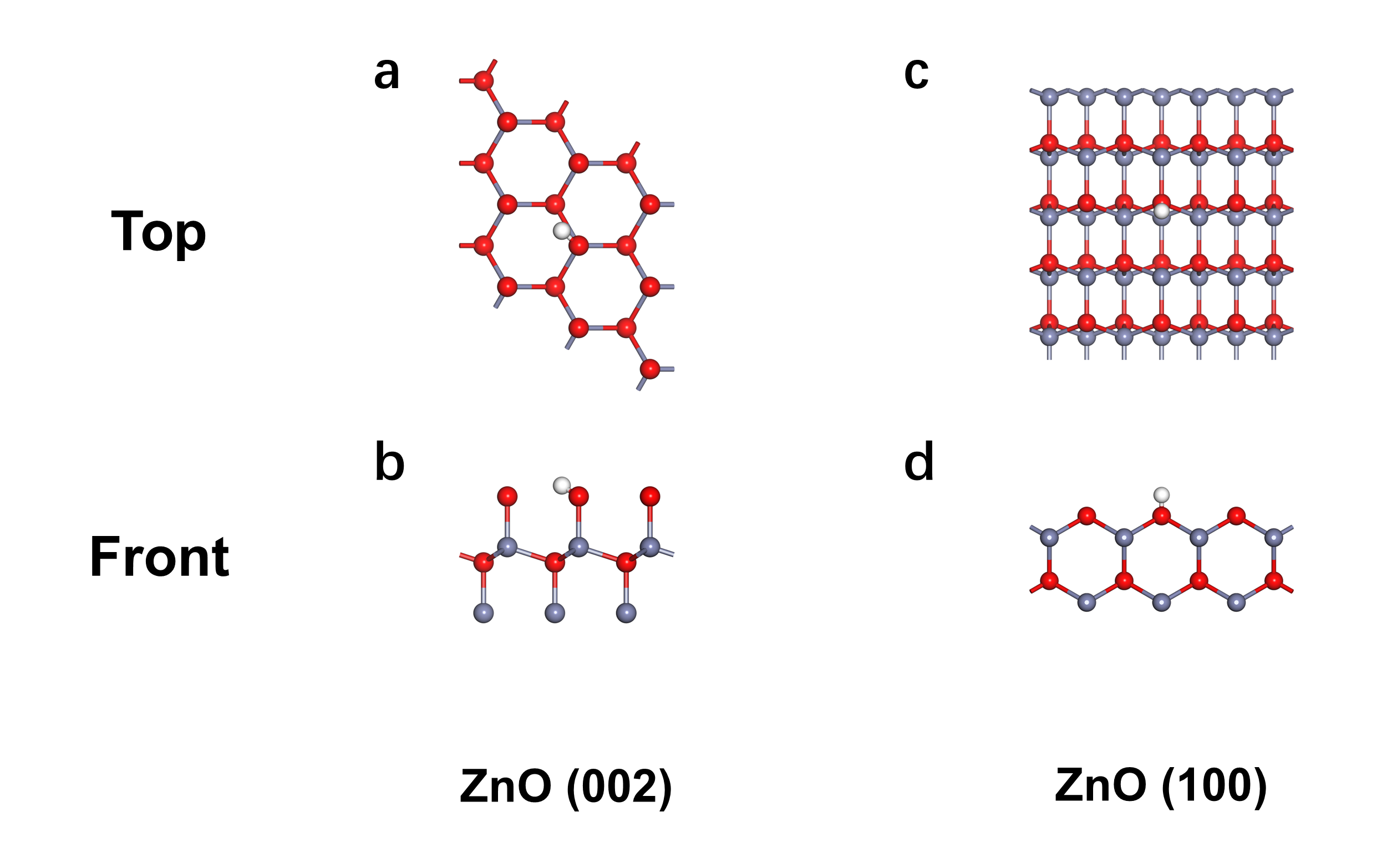


**Fig. S33** The adsorption sites of hydrogen proton in ZnO (002) and ZnO (100) lattices.


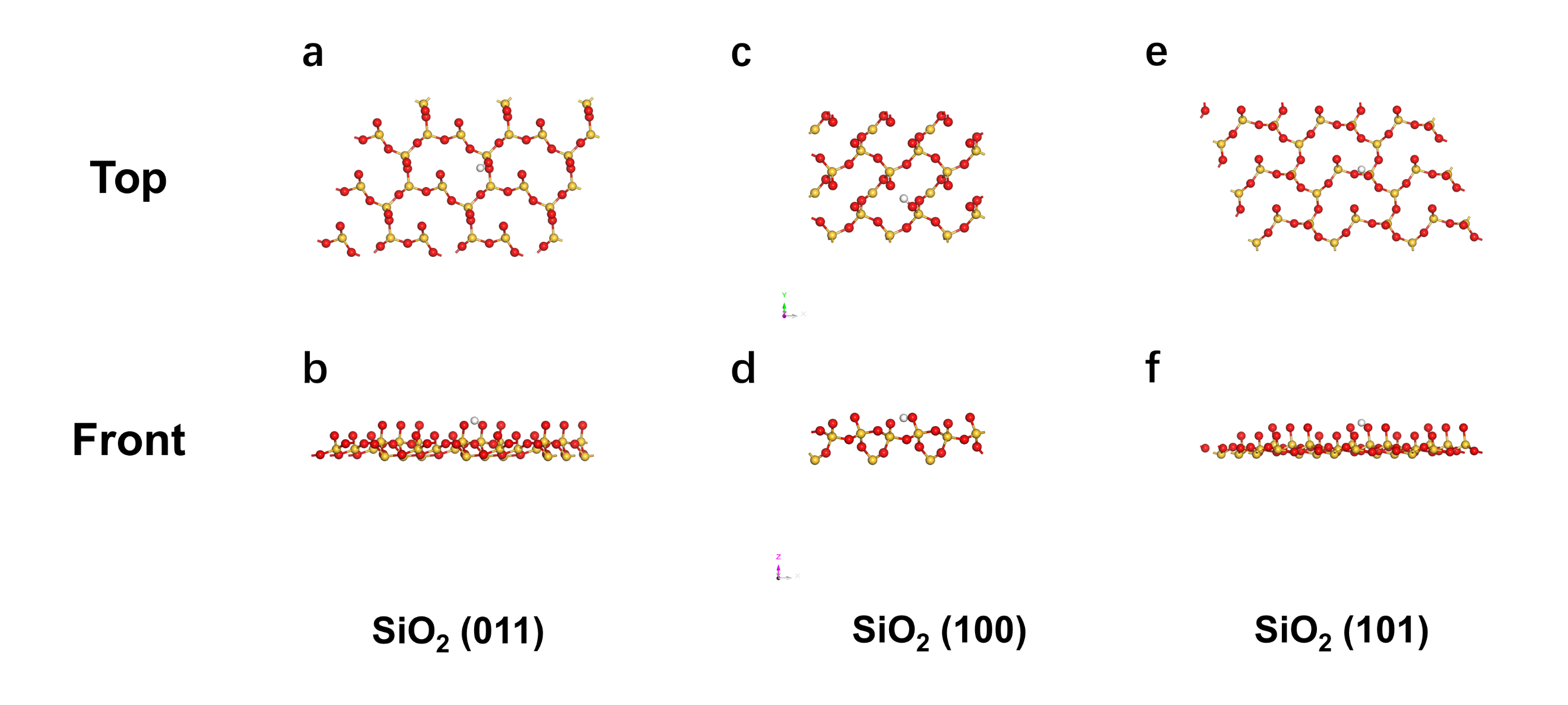


**Fig. S34** The adsorption sites of hydrogen proton in SiO_2_ (011), SiO_2_ (100) and SiO_2_ (101) lattices.


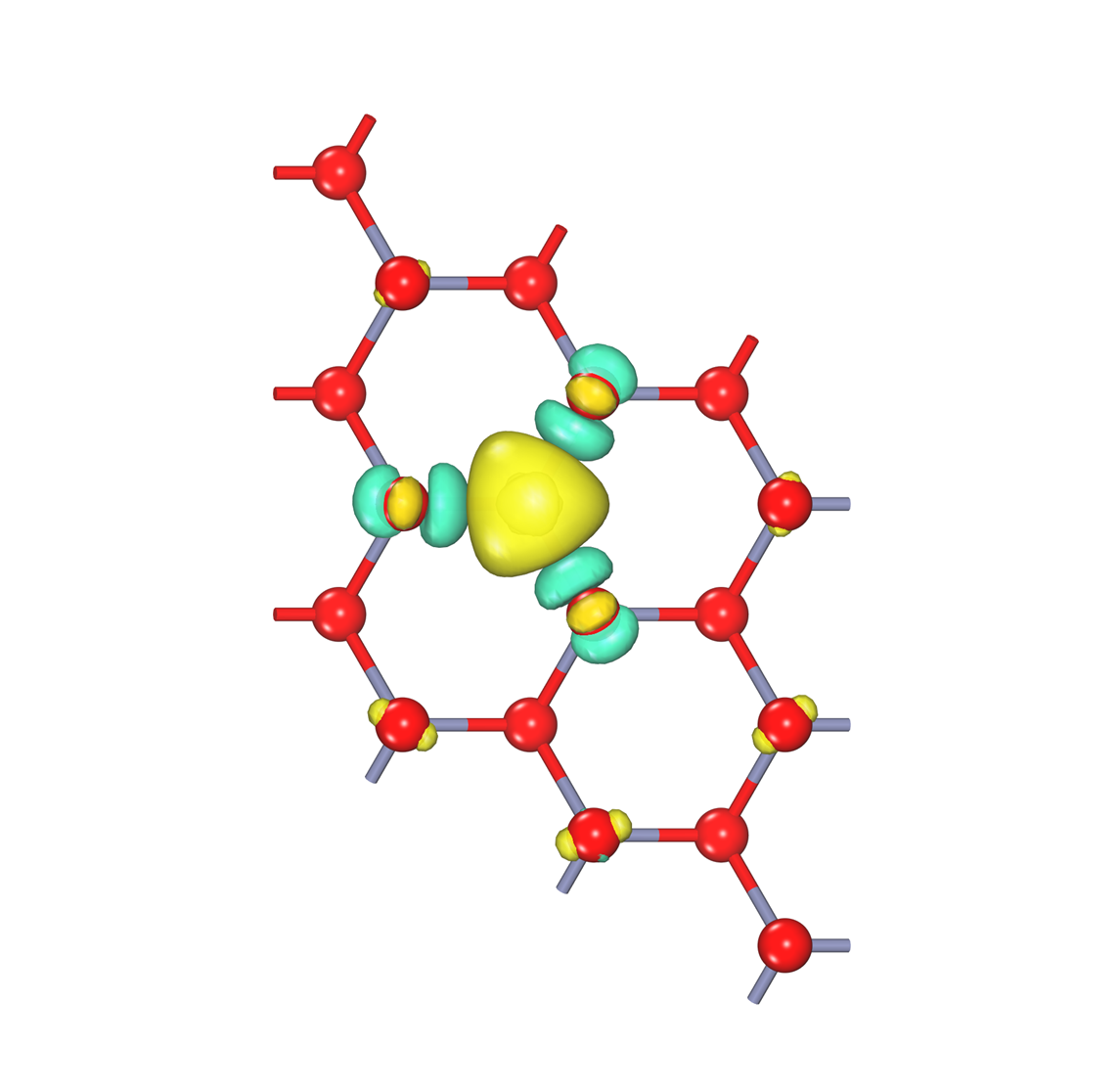


**Fig. S35** The charge distribution and of the electron density difference map at the ZnO interphase (top view).


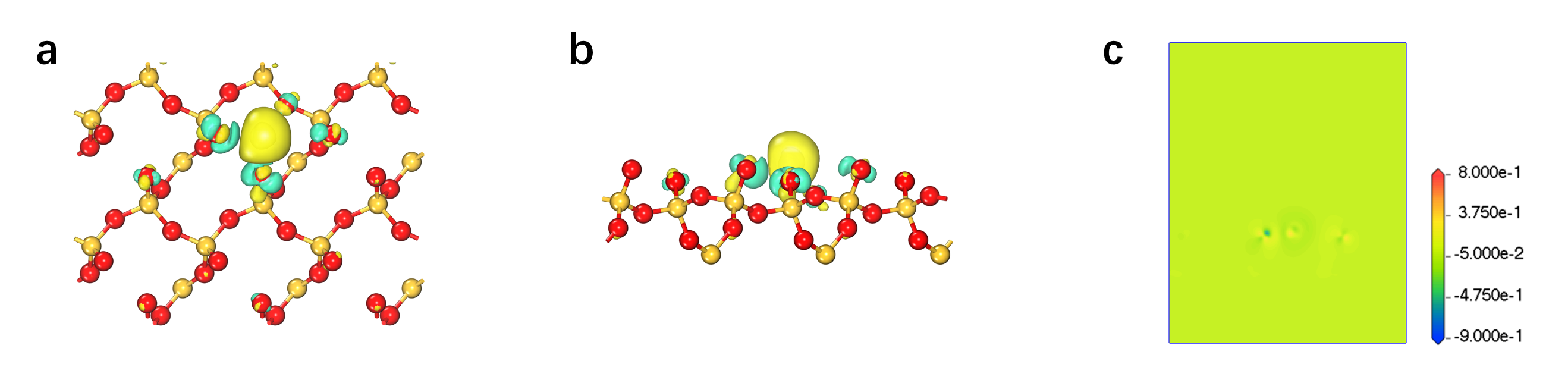


**Fig. S36** (a,b) The charge distribution and (c) the corresponding slice of the electron density difference map at the SiO_2_ interphase.





**Fig. S37** XRD patterns of SPC.


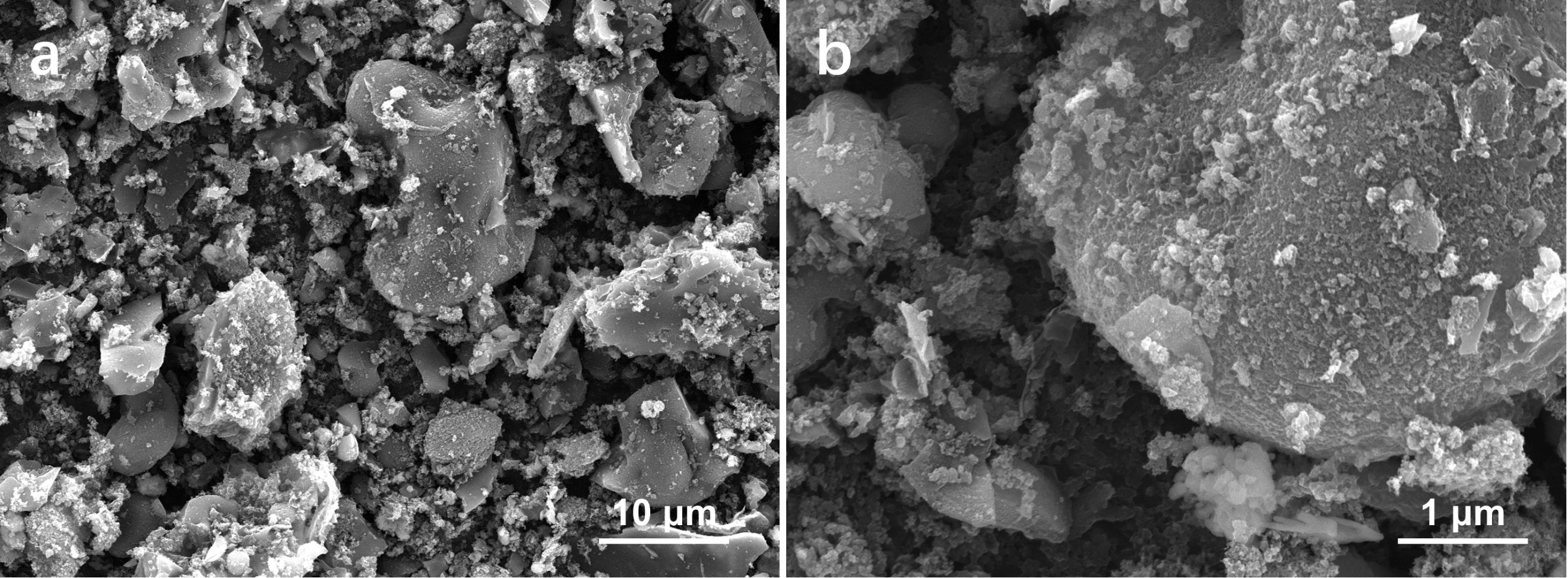


**Fig. S38** SEM images of SPC.





**Fig. S39** The slopes of impedance and low frequency of full cells.





**Fig. S40** The GCD curves of Zn//SPC full cells.


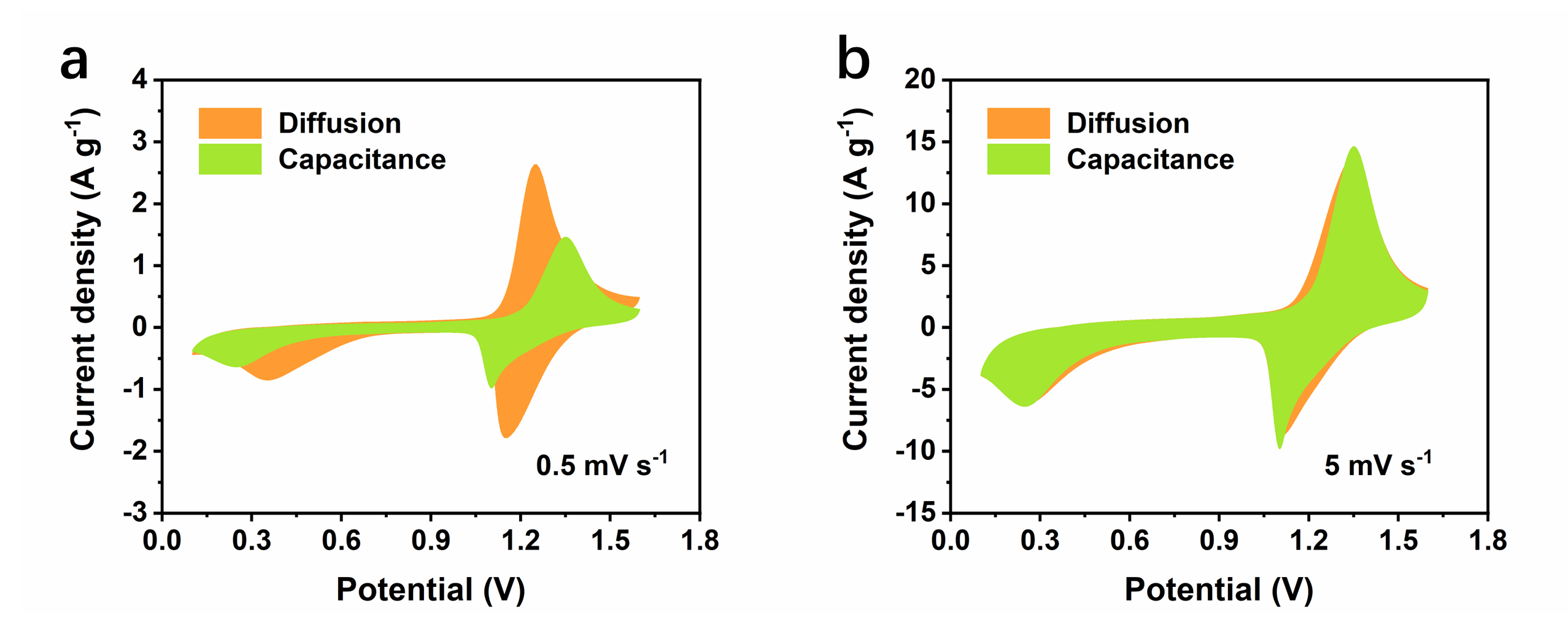


**Fig. S41** The diffusion/capacitance contribution ratio of Zn@ZSCP//SPC full cells varies as a function of scan rate.





**Fig. S42** The self-discharge test of Zn//SPC.





**Fig. S43** The long-term cycling stability of the full cells at 0.2 A g^-1^.

**References**

[1] D. Kundu, S. H. Vajargah, L. Wan, B. Adams, D. Prendergast, L. F. Nazar, *Energy Environ. Sci.* **2018**, *11*, 881.

[2] J. K. Nørskov, J. Rossmeisl, A. Logadottir, L. Lindqvist, J. R. Kitchin, T. Bligaard, H. Jonsson, *J. Phys. Chem. B* **2004**, *108*, 17886.
